# Evolutionary, structural and functional analysis of the caleosin/peroxygenase gene family in the Fungi

**DOI:** 10.1186/s12864-018-5334-1

**Published:** 2018-12-28

**Authors:** Farzana Rahman, Mehedi Hassan, Abdulsamie Hanano, David A. Fitzpatrick, Charley G. P. McCarthy, Denis J. Murphy

**Affiliations:** 1 Genomics and Computational Biology Research Group, University of South Wales, Pontypridd, CF37 1DL UK; 2 Department of Molecular Biology and Biotechnology, Atomic Energy Commission of Syria, P.O. Box 6091, Damascus, Syria; 3 Maynooth University, County Kildare Maynooth, Ireland

**Keywords:** Caleosin, Evolution, Fungi, Lipid droplets, Oxylipins, Peroxygenase, Stress responses, Viridiplantae

## Abstract

**Background:**

Caleosin/peroxygenases, *CLO/PXG*, (designated PF05042 in Pfam) are a group of genes/proteins with anomalous distributions in eukaryotic taxa. We have previously characterised *CLO/PXGs* in the Viridiplantae. The aim of this study was to investigate the evolution and functions of the *CLO/PXGs* in the Fungi and other non-plant clades and to elucidate the overall origin of this gene family.

**Results:**

*CLO/PXG*-like genes are distributed across the full range of fungal groups from the basal clades, Cryptomycota and Microsporidia, to the largest and most complex Dikarya species. However, the genes were only present in 243 out of 844 analysed fungal genomes. *CLO/PXG*-like genes have been retained in many pathogenic or parasitic fungi that have undergone considerable genomic and structural simplification, indicating that they have important functions in these species. Structural and functional analyses demonstrate that CLO/PXGs are multifunctional proteins closely related to similar proteins found in all major taxa of the Chlorophyte Division of the Viridiplantae. Transcriptome and physiological data show that fungal *CLO/PXG*-like genes have complex patterns of developmental and tissue-specific expression and are upregulated in response to a range of biotic and abiotic stresses as well as participating in key metabolic and developmental processes such as lipid metabolism, signalling, reproduction and pathogenesis. Biochemical data also reveal that the *Aspergillus flavus* CLO/PXG has specific functions in sporulation and aflatoxin production as well as playing roles in lipid droplet function.

**Conclusions:**

In contrast to plants, *CLO/PXGs* only occur in about 30% of sequenced fungal genomes but are present in all major taxa. Fungal CLO/PXGs have similar but not identical roles to those in plants, including stress-related oxylipin signalling, lipid metabolism, reproduction and pathogenesis. While the presence of CLO/PXG orthologs in all plant genomes sequenced to date would suggest that they have core housekeeping functions in plants, the selective loss of *CLO/PXGs* in many fungal genomes suggests more restricted functions in fungi as accessory genes useful in particular environments or niches. We suggest an ancient origin of *CLO/PXG*-like genes in the ‘last eukaryotic common ancestor’ (LECA) and their subsequent loss in ancestors of the Metazoa, after the latter had diverged from the ancestral fungal lineage.

**Electronic supplementary material:**

The online version of this article (10.1186/s12864-018-5334-1) contains supplementary material, which is available to authorized users.

## Background

Caleosin/peroxygenases (designated PF05042 in Pfam, or IPR007736 in InterPro) are an unusual group of genes/proteins with anomalous distributions in eukaryotic taxa. Caleosin/peroxygenase (*CLO/PXG*)-like genes are found almost universally in land plants (Embryophyta) and green algae (Chlorophyta) and are also found widely in all fungal groups but are almost completely absent from other eukaryotic groups such as protozoans and animals [1–4]. There are also numerous reports of the various physiological roles of the encoded CLO/PXG proteins in several Ascomycete and Basidiomycete fungal species [5–11].

The widespread distribution of *CLO/PXG*-like genes in just plant and fungal genomes is anomalous because the fungi are grouped with animals (Metazoa) as members of the monophyletic Opisthokont clade [12]. In contrast, land plants and green algae are grouped in a completely separate monophyletic eukaryotic lineage termed the Viridiplantae. If caleosins were present in the common ancestor of the Opisthokonts and Viridiplantae, the so-called ‘last eukaryotic common ancestor’ (LECA), then their presence would also be expected in at least some Metazoan genomes. That is unless these genes were lost prior to the earliest divergence of Metazoan groups, which probably occurred > 650 million years ago (Mya) [13–16]. Given the strong support for Metazoan monophyly [13, 14, 17], the loss of *CLO/PXG* genes early in protozoan evolution, i.e. before the appearance of metazoans, may be the most parsimonious explanation of the otherwise anomalous distribution of this gene family in eukaryotes.

*CLO/PXG*-like sequences are variously annotated in genome databases, such as NCBI and MycoCosm, as ‘caleosin’ and/or ‘peroxygenase’ although some are labelled variously as ‘hypothetical protein’, ‘ABA-induced protein’, calcium-binding protein’ or ‘EF hand protein’. In view of their widespread designations as either caleosins (*CLO*) and/or peroxygenases (*PXG*) we refer to these genes herein as *CLO/PXG*. In both plants and fungi, CLO/PXG proteins have been shown to have peroxygenase activities and are designated as members of the EC:1.11.2.3 class of oxidoreductases [3, 18–21]. Several different types of lipid peroxygenase activity are catalysed by both fungal and plant CLO/PXGs and in all cases these activities require the presence of heme groups that are coordinated by two invariant histidine residues [3, 18–22]. Lipid peroxygenases such as CLO/PXG play key roles in the metabolism of oxylipins, which are a large family of oxygenated fatty acids, and also of their derived metabolites that are implicated in plant-fungal crosstalk in both pathogenic and symbiotic associations [23].

The proteins encoded by *CLO/PXG* genes are typically of relative molecular mass 25–30 kDa and are characterised by a highly conserved single calcium-binding EF hand motif, a lipid-binding domain that often includes a proline-rich motif, and the two invariant heme-coordinating histidine residues required for peroxygenase activity [2, 20, 24–27]. CLO/PXG sequences also contain several predicted kinase sites including one group that is proximal to the C terminus [26–29]. Taken together these are the diagnostic features that make up the canonical motifs used to classify CLO/PXG proteins in databases.

An important structural feature of CLO/PXG proteins is the presence of one or more lipid-binding sites, the most conserved of which is located immediately adjacent to the calcium-binding, EF hand motif [4]. It has been shown that CLO/PXG isoforms from both plants and fungi are able to bind to several different subcellular membrane systems, including the ER and plasmalemma, and that this binding is mediated via a single transmembrane domain located close to the calcium-binding, EF hand motif [1, 2, 24, 30]. In addition to their associations with bilayer membranes in cells, many CLO/PXG isoforms are also able to bind in a highly stable fashion to the phospholipid monolayer membrane that surrounds intracellular lipid droplets (LDs). The LD binding may be mediated by the relatively hydrophobic motif that is normally described as a transmembrane domain (see above), although such binding would require that the normally linear α-helical region could also assume a U-shape so that it could loop into and out of the LD monolayer [1, 31]. It has been proposed that the U-shape conformation of the lipid binding site when interacting with LDs is mediated by a relatively well-conserved proline-rich motif that is similar to the U-shaped LD-binding domain of oleosin proteins in plants [31–34].

In the case of plants and algae, CLO/PXGs have been shown to be involved in a wide range of physiological functions, including drought and osmotic stress responses [35–38], pathogen responses [39, 40], toxin formation, transport and sequestration [41], stomatal regulation in leaves, water transpiration, seed germination and G protein signalling [42], nitrogen deprivation [30, 32, 43–45], and dark adaptation [46]. In seeds and pollen grains, CLO/PXGs have also been shown to have roles in lipid packaging and mobilization [30, 31, 47–49]. In addition, the lipid peroxygenase activity of CLO/PXGs in plants is associated with epoxy fatty acid biosynthesis as part of oxylipin metabolism [20, 22, 50] as well as a broader series of epoxidation, hydroxylation and aromatization activities on substrates including terpenes and acyl derivatives [51].

In this study, we have performed a systematic analysis of the *CLO/PXG* gene family across 844 sequenced fungal species and compared these genes and their encoded proteins with their recently described orthologs from the Viridiplantae in terms of their motif architectures, secondary structures, physiological functions and possible evolutionary origins in eukaryotes [4]. We also aim to elucidate the wider functions of *CLO/PXG* gene family with a special focus the possible roles of CLO/PXGs in developmental and pathogenesis-related processes in fungi.

## Methods

### Data sources

The major fungal CLO/PXG data were retrieved from the following public repositories: NCBI (http://ncbi.nlm.nih.gov/), FungiDB (fungidb.org/), MycoCosm from JGI genome portal (https://genome.jgi.doe.gov/programs/fungi/index.jsf) and Ensembl Fungi portal (https://fungi.ensembl.org/index.html). Details of the various bioinformatics tools and packages used in this study, including web links, are listed in Additional file 1: Table S1. The accession number(s) of the analysed data is mentioned in the availability of data section.

### Finding and assessing representative CLO/PXG sequences

To find and assess candidate CLO/PXG sequences across all of the fungal species, previously identified CLO sequences of *Aspergillus flavus* (AflCLO), *Erysiphe necator* (EnCLO), *Neurospora crassa* (NcCLO), *Magnaporthe oryzae* (MoCLO), *Beauveria bassiana* (BbCLO), *Ustilago maydis* (UmCLO), *Rhodotorula toruloides* (RtCLO), *Gonapodya prolifera* (GprCLO), *Rhizophagus irregularis* (RiCLO), *Allomyces macrogymus* (AmaCLO), *Rozella allomycis* (RaCLO) were obtained from NCBI (http://www.ncbi.nlm.nih.gov/) via local BLASTp searches [52, 53]. Additionally, we retrieved a comprehensive list of 844 fungal species from the JGI MycoCosm genome portal. This list worked as a master copy to identify possible presence or absence of CLO/PXGs in fungal species. Using the reference genomes, fungal species from our master copy and by comparing the proteomes available in public databases, we accumulated a comprehensive list of CLO/PXG genes in fungal species.

In order to find CLO/PXG sequences across fungal species, we used the reference species sequences to perform a comprehensive BLASTp search using NCBI’s BLAST+ toolset. Using the following parameters the BLASTp search resulted with > 1200 sequences: maximum target sequence: 100, expected threshold: 13, word size: 10, scoring matrix: BLOSUM62, gap cost: existence 11 and extension 1, compositional adjustments: conditional compositional score matrix adjustment. The resulted sequences were further analysed using InterProScan (http://www.ebi.ac.uk/interpro/) to confirm the presence of caleosin specific ‘calcium-binding EF-hand motif’. Additionally, visual inspection was conducted using CLC genomics workbench version 10.0.3 to confirm the presence of canonical CLO/PXG domains. After comparing the retrieved data using peer-reviewed toolsets and analysing motifs, physical and chemical properties of the candidate sequences, presence of 344 CLO/PXG sequences was confirmed across 243 species. A full list of the 844 fungal species analysed to determine the presence and absence of CLO/PXG is shown in Additional file 2: Table S2.

Based on the quality of sequenced genomic data, we identified 243 species from the kingdom Fungi where their genomes contained very high probability CLO/PXG gene sequences. In contrast, the remaining 601 fungal species that were analysed did not show the presence of CLO/PXG sequences according to the data available on public repositories. The identified species were representatives from all major fungal groups, which included Basidiomycota, Ascomycota and basal or *incertae sedis* clades (Mucoromycota, Zoopagomycota, Blastocladiomycota, Chytridiomycota, Cryptomycota and Microsporidia). Even though the Ascomycota and Basidiomycota are the source of the major available sequenced data, we are confident that there is sufficient genomic data from the sequenced non-Ascomycota and non-Basidiomycota groups to elucidate the evolution of CLO/PXG gene family in the Fungi.

### Analysis of physical and chemical properties

In order to collect comprehensive information about CLO sequences, physiological properties of each caleosin were analysed. To identify each CLO sequence individually, both the NCBI accession and pfam accession numbers were retrieved from their respective databases. Using the Henderson-Hasselbalch equation, the isoelectric point of each sequence was computed [54]. Physical and chemical properties of each CLO sequence such as amino acid composition, molecular weight and isoelectric points were computed using protein identification and analysis tool, ExPASy [55]. The physiological properties of CLO sequences, number of CLO per sequence, and NCBI accession number are shown in Additional file 3: Table S3. For more detailed analysis, we selected 40 representative sequences from all of the major fungal groups out of 344 CLO/PXG sequences originally identified (Additional file 4: Table S4).

### Sequence analysis

#### Identifying motifs

**Fig. 1:**
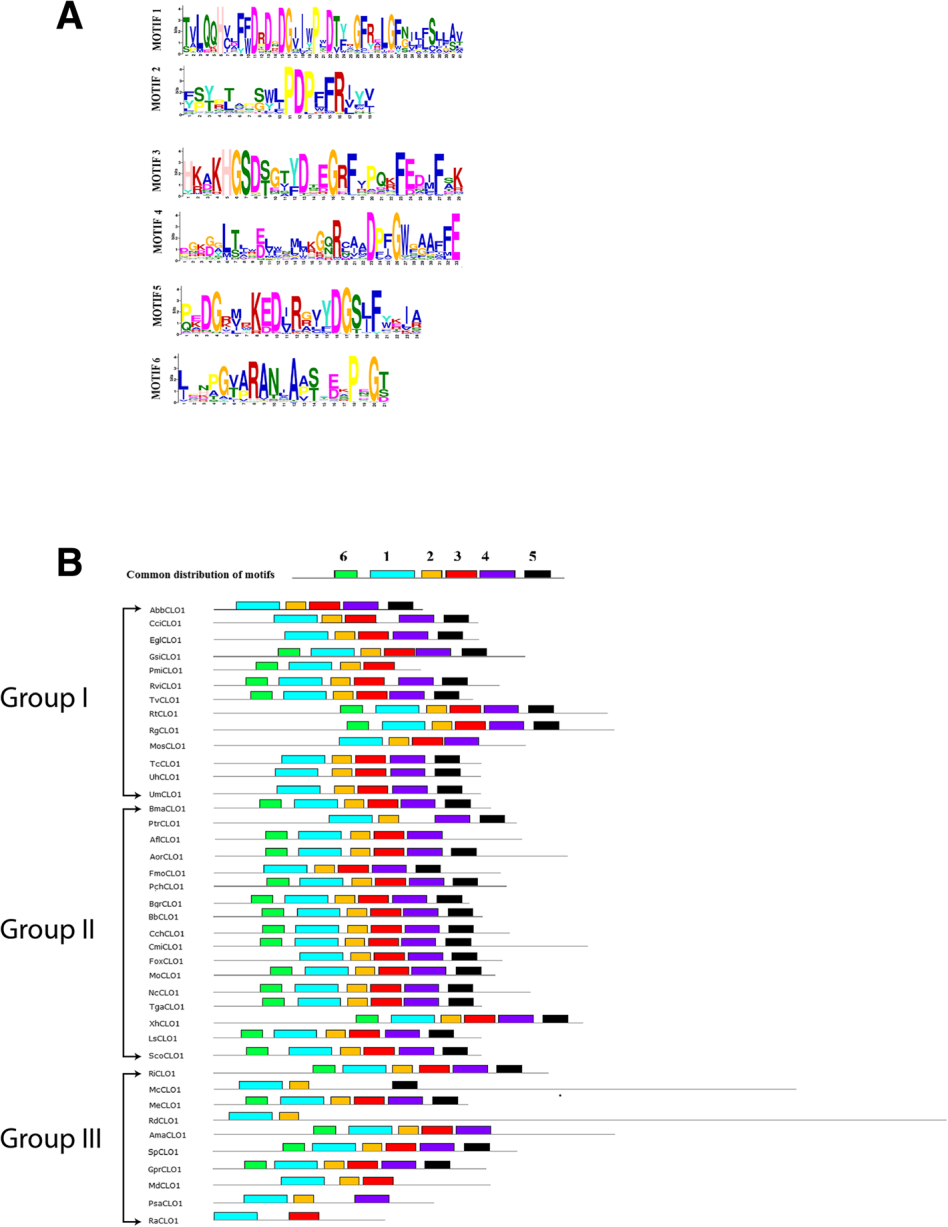
Motif analysis of 40 selected fungal CLO/PXG sequences from each of the major phyla. **a** six motif logo generated from the consensus amino acid sequences; **b** distribution of the motifs in the 40 selected fungal species

**Fig. 2:**
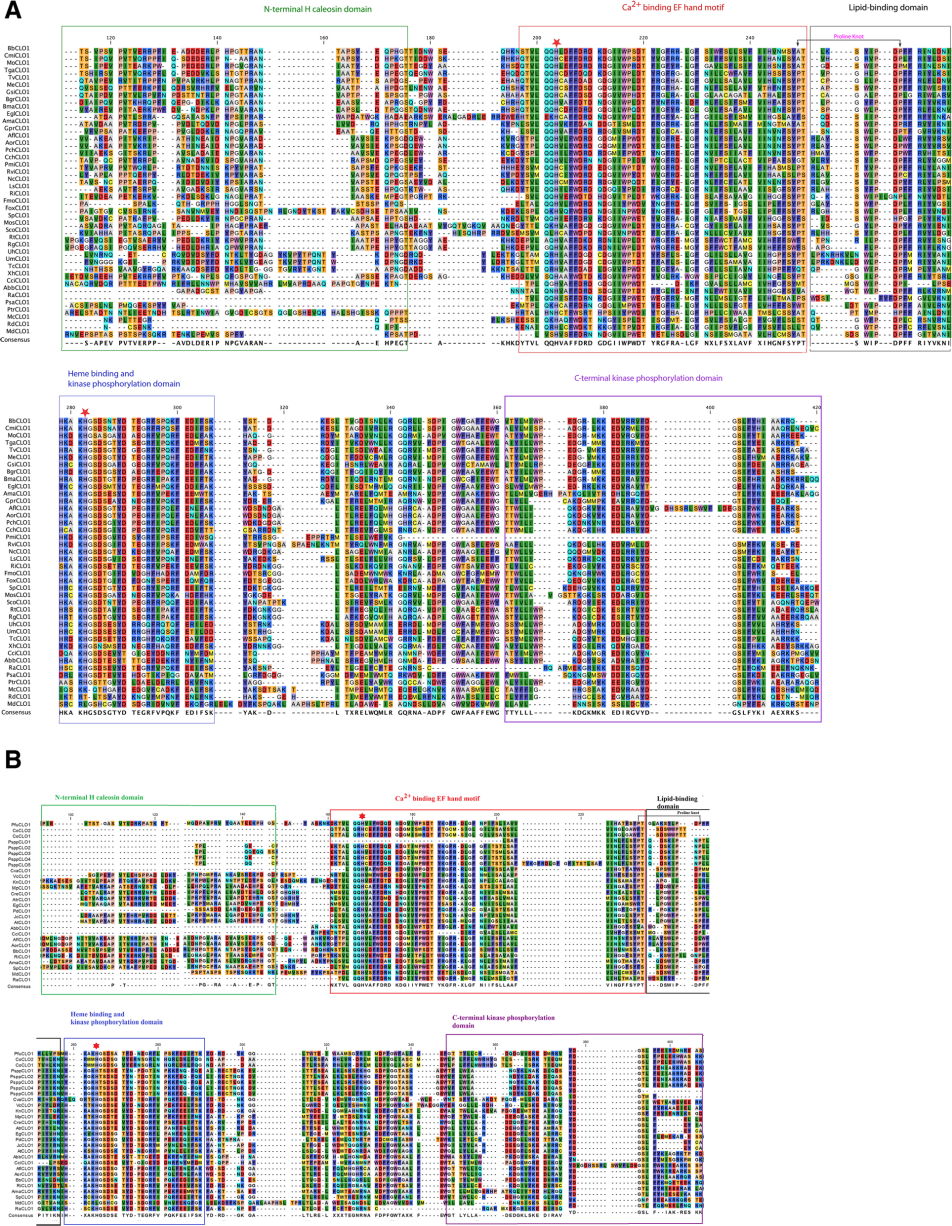
CLO/PXG protein sequence alignments from representative fungal species. **a** 40 selected fungal species showing the major structural and functional domains; **b** 23 sequences from non-Dikarya species. The major conserved domains are shown in boxes: N-terminal H-domain, Ca^2+^ binding EF hand, lipid-binding domain, haem binding and kinase domain, C-terminal phosphorylation domain. The two near-invariant haem-coordinating histidine residues are shown with a red star

The CLO/PXG sequences were analysed to identify amino acid motifs in order to identify conserved domains that are known to contribute to the biological activities of these proteins. For motif discovery, the selected 40 caleosin sequences were analysed using the expectation maximization technique [56, 57]. The MEME software package was utilised to assess the motifs across species [56, 57]. Using the discriminative mode and a window size of minimum 15 and maximum 50, each CLO sequence was analysed to identify motifs. We identified distinctive six motifs, which we and others have recently shown to be associated with important biological functions and activities across plant species [4]. The distinctive motif logos along with their distribution pattern of the selected 40 sequences across species are shown in Fig. 1. The full CLO/PXG protein sequence alignments showing the major structural and functional domains from 40 selected species from throughout the fungi plus 23 sequences from non-Dikarya fungal species are presented in Fig. 2.

#### Transmembrane domain and secondary structure prediction

**Fig. 3:**
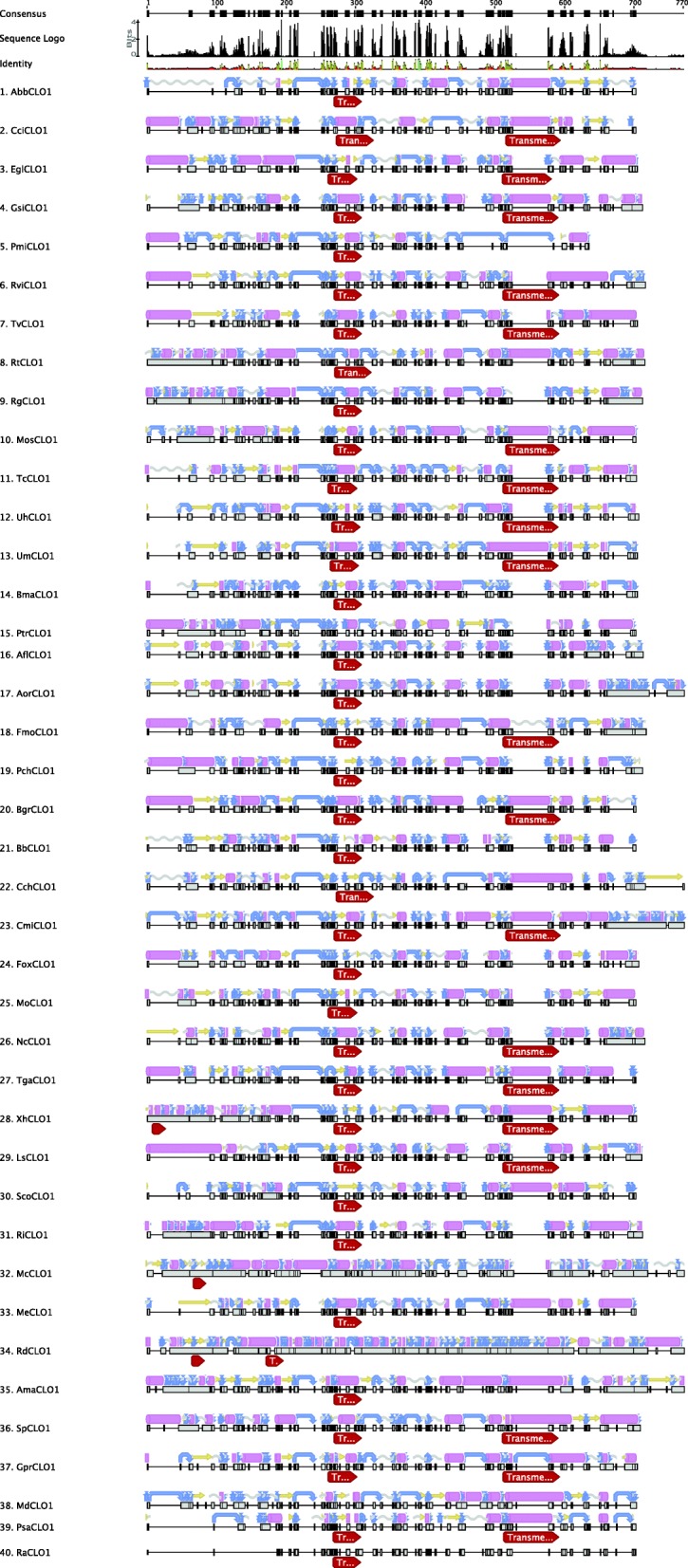
Predicted secondary structures and TM of 40 selected fungal CLO/PXG proteins. The red right hand arrow showing the transmembrane domain for each sequence. For secondary structure, the annotation goes as: pink tube shows Alpha helix, the yellow arrow shows beta strand, blue arrow shows turn, grey coil shows the coil of each sequence

In order to identify putative membrane-spanning and/or lipid-binding regions, transmembrane predictions were conducted using Geneious package version 11.1.5. The secondary structure for each CLO sequence was observed using the secondary structure prediction tool of Geneious. To prepare the sequences for transmembrane and secondary structure prediction, multiple sequence alignments were performed using the built-in Geneious alignment and ClustalW alignment of the Geneious package. The following parameters were used in both alignment method to align the protein sequences a) Cost matrix BLOSUM 45, b) Gap open penalty 12, c) Gap extension penalty 3, and d) refinement iteration of 2. The location (start and end) of each transmembrane domain is shown in Additional file 5: Table S5. The lengths of the predicted transmembrane regions are consistent at 21 residues. The predicted localisations of transmembrane regions in the selected 40 species is shown in Fig. 3 and the full predicted transmembrane regions and secondary structures of 344 sequences are shown in Additional file 15: Figure S3A.

#### Gene structure prediction

**Fig. 4:**
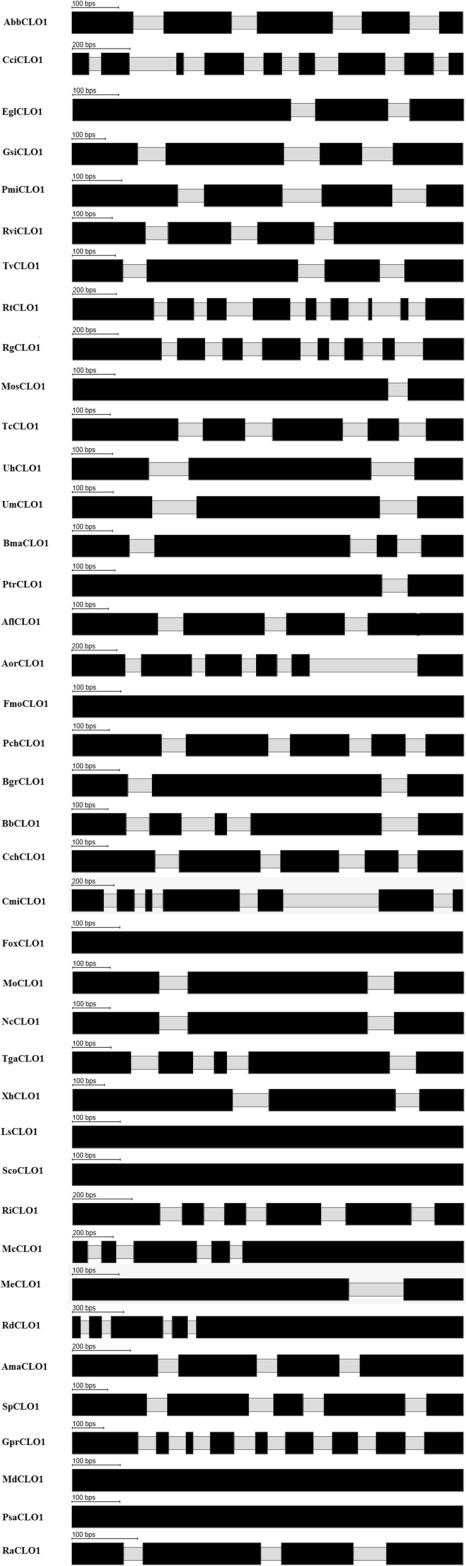
Predicted gene structure of 40 representative fungal *CLO/PXG*s. The prediction shows the locations of introns (grey) and exons (black). Note that the gene lengths are quite variable so for clarity they have all been scaled to the same lengths here

In order to identify the number of intron-exon per species sequence, the Scipio program based on BLAT alignment was used to determine the gene structures [58]. For gene structure prediction, genome sequences that correspond to all of the 344 protein sequences were retrieved from NCBI and FungiDB. Using Scipio program version 1.4, each protein sequence was scanned against the corresponding genome sequence to identify the intron-exon number and location. To visually inspect the gene structure, each result file from Scipio program was uploaded to the web version of Scipio program commonly known as webscipio. The resulting intron-exon structures of the selected 40 fungal CLO/PXG sequences are shown in Fig. 4. The following steps explain the gene structure prediction pipeline utilised for our study.

- CLO/PXG protein sequence collection: From the combined list of CLO/PXG sequences, separate each sequence and make individual CLO/PXG sequence fasta file and give unique identifier as file name (e.g. *M. circinelloides* protein fasta was named as McCLO1.protein.fasta)
- Genome sequence collection: For each CLO/PXG sequence, collect the corresponding whole genome sequence from the NCBI genome FTP site (ftp://ftp.ncbi.nlm.nih.gov/genomes/).
- Curate the collected genomic dataset to remove redundant sequences
- Create individual genome fasta files for each corresponding CLO/PXG sequence file and give it unique name (e.g. *M. circinelloides* genome sequence was named as Mc1.genome.fasta)
- Map each protein fasta file to corresponding genome fasta files using scipio command line interface. In this step several output files were created, from which files (extension with .yml) containing intron-exon information were the files of interest.
- Parse data from output files to separate intron-exon data.
- Process each intron-exon data file using WebScipio interface to get intron-exon image file.
- Process individual image files and gather intron-exon data in one file for further analysis.

#### MSA (multiple sequence alignment)

Multiple sequence alignment and domain analyses were performed using ClustalOmega software, version 1.2.2, due to its improved scalability over previous Clustal versions and its ability to produce accurate alignments in a relatively short time [59, 60]. To generate highly accurate alignments, mBed-like clustering guide tree and iteration parameters were selected as ‘yes’. The conserved domains were identified using the Uniprot domain analysing utilities [61]. The alignments were inspected using the CLC Genomics Workbench 10.0.3 (https://www.qiagenbioinformatics.com/). Complete alignments with RasMol colour codes are shown in Fig. 2.

### Phylogenetic analyses

Pfam (Protein families) domain prediction was performed for 462 plant and fungal CLO/PXG proteins using InterProScan, and predicted CLO/PXG domains were extracted from each protein sequence. All 462 CLO/PXG domains were aligned using MUSCLE with the default parameters, and the best-fit evolutionary model was predicted for the alignment using ProtTest [62, 63]. A maximum-likelihood phylogenetic reconstruction of the 462 domains was performed using PhyML with a LG + G evolutionary model and 100 bootstrap replicates [64]. In addition, a taxonomically representative dataset containing 199 fungal and plant CLO/PXG domains was extracted from the full dataset and a phylogeny was generated for this dataset using the same procedure above (with LG + G also predicted as the best-fit evolutionary model). Both phylogenies were visualized using iTOL and annotated with CLO/PXG motif data as determined by MEME [56, 57, 65, 66]. A neighbour-joining network of phylogenetic splits within the representative dataset alignment was generated using SplitsTree [67]. The statistical confidence of the representative phylogeny was assessed by comparing its likelihood to a phylogeny that grouped by species taxonomy and also 100 randomized phylogenies. Site-wise likelihood data for each phylogeny was calculated using tree-puzzle and assessed using Consel, specifically the approximately unbiased test of phylogenetic tree selection [68, 69].

### Wet lab materials, chemicals, strain, culture conditions and treatments

Primers were purchased from either Eurofins or Sigma-France. Aniline, thiobenzamide, cumene hydroperoxide and aflatoxin B1 were purchased from Sigma-Aldrich, Germany. The *Aspergillus flavus* strain NRRL3357 kindly provided by the Faculty of Agricultural Sciences, Gembloux, Belgium. Stock cultures of *A. flavus* were routinely maintained in slant tubes at 4 °C on potato dextrose agar (PDA) (Difco Laboratories, USA). For solid or liquid cultures of *A. flavus*, stock cultures were transferred onto Petri dishes containing PDA or into a 500-mL Erlenmeyer flask containing 100 mL of PD broth and allowed to develop for 7 days at 28 °C. Total oxylipins were extracted from maize seedlings according to [70]. In brief, five grams of plant material were immediately ground in liquid nitrogen and hydrated with 5 mL of extraction solvent (*n*-hexane:2-propanol:3/2) (*v*/v). The mixture was ultra-homogenized for 3 × 30 s on ice, left on a shaker for 10 min and centrifuged at 3000×*g* at 4 °C for 10 min. The upper phase was filtered through 5 g of sodium sulfate and dried under nitrogen. Finally, the oxylipin extract was dissolved in 100 μL ethanol and stored at − 20 °C until use. An ethanol solution of extracted oxylipins (50 and 100 μM) was added to the liquid cultures of *A. flavus* in a 200-mL Erlenmeyer flask containing 50 mL of PD broth for 2 days at 28 °C. Subsequently equal quantities of fungal material were transferred onto PDA-plates that were surface-covered with 50 or 100 μM of extracted oxylipins and allowed to develop for 7 days at 28 °C. This experiment was done in triplicate. In parallel, a control experiment was carried out using ethanol only. Peroxygenase activity was routinely measured by hydroxylation of aniline as substrate [71].

### Measurements of fungal biomass and conidia number

Fungal biomass was estimated on 7-day old PDA-plate cultures. A single point inoculation was done onto a cellophane membrane placed on the surface of a PDA plate. The membranes were removed and the mycelia were carefully taken, washed twice thoroughly with distilled water, filtered through Whatman no. 4 filter papers and dried in an oven at 95 °C for overnight. The weights of the dried mycelia were then determined according to Rasooli and Razzaghi-Abyaneh [72] . The total conidia for each plate was harvested and suspended in 5 ml of water containing 0.01% Tween 80. Diluted to 1:10, conidia were counted with a hemocytometer.

### Gene transcript analysis

For a complete list of nucleotide sequences of primers used in this study please refer to [3]. *A. flavus* was grown as described above and the total fungal RNAs were isolated using an RNeasy kit according to the manufacturer’s instructions (Qiagen, Germany). DNA-free RNAs samples were diluted to 50 ng L^− 1^ using RNase-free water and stored at − 80 °C. Subsequently, the respective cDNAs were synthesized using M-MLV RT (Invitrogen) as described previously [2]. Gene transcripts were evaluated by RT-qPCR using an AriaMx Real-time PCR System (Agillent technologies, USA). The 25 μl reaction mixtures contained 0.5 μl of each of the target and reference gene primers, 12.5 μl of SYBR Green qRT-PCR mix (Bio-Rad, USA) and 2.5 μl of 10-fold diluted cDNA. RT-qPCR conditions were as previously described [2]. Each point was triplicated and the average of *C*_T_ was taken. The relative quantification RQ _=_ 2^(−∆∆*C*T)^ of the target gene was determined using the software of an Agillent AriaMx Real-time PCR System.

### Extraction, clean-up and HPLC analysis of aflatoxin

The extraction of aflatoxin (AF) produced by *A. flavus* was carried out on the total fungal growth according to Bertuzzi et al., 2011 [73] using 100 mL of chloroform during one hour on a rotary-shaker. The extract was analysed on TLC plate as described by [74]. For this, extracted AF samples were spotted onto a C_18_ reversed-phase TLC plate (Aluminium sheets 20 × 20 cm, 200 μm layer, Merck, Germany) and the chromatogram was developed using a solvent system of chloroform/acetone (90:10, *v*/v). After migration, the spot having a *Rf* value similar to aflatoxin B1 (AFB1) standard was scraped off, re-extracted with chloroform and evaporated to dryness under nitrogen. The extract was dissolved in 100 μL acetonitrile and stored in amber-coloured vials at + 4 °C. Extracts were analysed using a Jasco LC-2000 plus series HPLC system (Jasco, USA) using a fluorescence detector (RF-10Axl, Shimadzu) (λexc 247 nm; λem 480 nm) and a C18 column (Eclipse XDB-C18 150 × 4.6 mm, 5 μm; Agilent, USA, column temperature 53 °C). The run (10 min) was performed using a mobile phase of water/methanol/acetonitrile (50/40/10, v/v/v) at a flow rate of 0.8 ml min^− 1^ and a run time of 10 min.

### Statistical analysis

Data were expressed as means ± standard deviation (SD). Comparisons between control and treatments were evaluated by t-test. Difference from control was considered significant as *P* < 0.05, very significant as *P* < 0.01.

## Results

### *CLO/PXG*-like sequences are widespread but not ubiquitous in fungi

We retrieved a total of 844 sequenced fungal genomes from public databases (as of June 2018), of which 243 genomes (29% of the total) contained *CLO/PXG*-like sequences (Table 1).

**Table 1:** List of sequenced fungal genomes that contain CLO/PGX-like sequences

Phylum	Subdivision	Class	Sequenced genomes	Genomes with CLO-like seqs	Genomes with H-domains	% spp. with CLO-like seqs
Cryptomycota/ Rozellomycota			2	2	0	100
Microsporidiomycota			9	1	0	
Chytridiomycota			9	1	1	
Neocallimastigomycota			5	1	0	
Blastocladiomycota			4	2	2	
Zoopagomycota			15	1	1	
Mucoromycota			44	6	3	
Glomeromycota			1	1	3	
All non-Dikarya			89	15	10	16.9
Ascomycota			505	158		31
	Pezizomycotina		447	155		34.7
		Dothideomycetes	123	27		
		Eurotiomycetes	134	66		
		Lecanoromycetes	4	0		
		Leotiomycetes	29	8		
		Orbiliomycetes	2	0		
		Pezizomycetes	17	1		
		Sordariomycetes	135	55		
		Xylonomycetes	3	1		
	Saccharomycotina					
		Saccharomycetes	48	1		
	Taphrinomycotina		10	2		
Basidiomycota			252	69	34	27
	Agaricomycotina		214	52	29	24.3
		Agaricomycetes	196	48	29	
		Dacrymycetes	4	3	0	
		Tremellomycetes	14	1	0	
	Pucciniomycotina		15	6	4	40.0
		Microbotryomycetes	14	5	4	
		Mixiomycetes	1	1	0	
	Ustilaginomycotina		23	11	1	47.8
		Agaricostilbomycetes	1	0		
		Exobasidiomycetes	15	6	0	
		Tritirachiomycetes	1	0		
		Ustilaginomycetes	6	5	1	
All Dikarya			756	225		30.0
All Fungi			844	243		29.0

243 fungal genomes (29% of the total) from public databases contained *CLO/PXG*-like (as of June 2018) sequences

The full list of the 844 sequenced fungal genomes that were analysed by BLAST searches for the presence of *CLO/PXG*-like sequences is shown in Additional file 1: Table S1. The majority of *CLO/PXG*-containing fungal genomes harbour a single gene copy but some genomes have two copies and a few have between three and five copies (see Additional file 2: Table S2). Similarly to the *CLO/PXG* genes in plant genomes [4], these multiple copies in fungal genomes have most probably arisen via tandem repeats of gene segments, or less likely via duplication events from a single original gene. This is supported by the monophyletic nature of the ‘true’ Fungi, or Eumycota, which are a defined as a group of eukaryotic heterotrophs that reproduce with spores and have chitinous cell walls that probably diverged over one billion years ago [75, 76]. As shown in Table 1 and Additional file 3: Table S3, the list of currently sequenced fungal genomes is heavily biased towards agriculturally and medically important species within the superphylum, Dikarya, which make up > 89% of the total lodged in public databases. Out of the 755 sequenced genomes in the two Dikarya phyla, the Ascomycota and Basidiomycota, 31 and 27% of species respectively contain one or more well-supported *CLO/PXG*-like sequences.

In contrast, only 89 non-Dikarya species sequences were present in public databases. This is not because there are fewer species in the non-Dikarya but rather that they are generally less well studied and less likely to have significant economic impacts which means that fewer resources have been devoted to characterising their genomes. The non-Dikarya for which some sequence data are available include eight phyla ranging from the relatively advanced Mucoromycota to more basal taxa such as Chytridiomycota, Microsporidia and Cryptomycota where very few genomes have yet been sequenced. Of the 89 non-Dikarya species that we surveyed, only 15 contained *CLO/PXG*-like sequences (16.9% of the total). As discussed below, some of these sequences were relatively divergent and a few lacked some, but not all, characteristic features of the better-studied *CLO/PXG* sequences from Dikarya. However, the small sample size precludes any robust conclusions about the relative abundance of *CLO/PXG*-like sequences in these fungal phyla. Indeed it might be the case that, as more non-Dikarya species are sequenced, the proportion of genomes that harbour *CLO/PXG*-like sequences might rise to the approximately 30% level found in the Dikarya.

### Motif architecture of fungal CLO/PXG proteins

The Multiple En for Motif Elicitation (MEME) software package was used to generate a series of conserved CLO/PXG protein regions, or motifs, in all 344 of the identified fungal sequences (Additional file 10: Figure S1). Because this file is rather large for easy comparative analysis, we also generated a shorter list of the domain architecture of 40 representative species across all the major fugal groups as shown in Fig. 1. A full list of these 40 species is shown in Additional file 3: Table S3 where it can be seen that the list includes 13 spp. from Basidiomycota, 17 spp. from Ascomycota and 10 spp. from non-Dikarya phyla. In terms of setting the parameters for the MEME analysis, we found that the optimal number of motifs to give reproducible and meaningful results was with an upper limit of six. This is in contrast with our recent comprehensive analysis of CLO/PXG proteins in the Viridiplantae where optimal results were obtained by setting a limit of seven motifs [4]. Figure 1 and SI Fig. 1 show the highly conserved nature of the CLO/PXG motifs, both in terms of their amino composition and their position in the protein, across the entire fungal kingdom. The five main motifs, numbered from the N-terminus as 1–2–3-4-5, occur in the same order in the vast majority of the 344 analysed fungal CLO/PXG sequences.

In some sequences the MEME software failed to detect one or more of these motifs but manual inspection revealed that this was due to small changes in a few amino acids that resulted in a lack of recognition of the motif by MEME although its major features were in fact present and intact. For example, in the case of the basal species, *Rozella allomyces*, three of the five major CLO/PXG motifs appear to be missing according to MEME (final line in Fig. 1b), but manual inspection shows that the ‘missing’ motifs 2, 4 and 5 are essentially present, albeit in somewhat modified form compared to the other fungal sequences. Despite extensive trials, we were not able to find any better motif prediction software than MEME for this particular analysis but it is evident that even MEME is not always fully reliable. Nevertheless, the method is still useful for a broad-brush analysis of large numbers of sequences, with the proviso that manual curation is still required for more accurate results.

One example of a useful prediction from MEME was the discovery of a sixth motif, labelled 6 in Fig. 1b and Additional file 10: Figure SI, that was located close to the N-terminus of the proteins. This motif (shaded green in the gene name column in the above Figures) was present in 258 of the 344 fungal CLO/PXG sequences. Interestingly, fungal motif six was structurally similar to the N-terminal located motif 4 that we previously found in Viridiplantae sequences [4]. This motif is also referred to in the literature as the ‘H-domain’, which in plants is a 30–50-residue sequence that is found in most CLO/PXG isoforms in some algae and more primitive land plants [27, 29, 77, 78]. In contrast, we found that in the much more recently evolved angiosperm phylum (also known as Angiospermae or Magnoliophya), which includes all flowering plants, every genome contained at least one *CLO/PXG* gene copy with an H-domain plus one copy where the H-domain was missing – the latter are termed L-domain isoforms because the proteins are 3–5 kDa lighter than the corresponding H-domain isoforms [4]. In the case of angiosperms, the fact that each genome contains at least one copy of each isoform indicates that they probably have separate functions and it has been proposed that they may be located in different subcellular compartments [4]. In the Fungi, 86 of 344 the sequenced *CLO/PXG* genes encode proteins that lack motif 6 and are therefore analogous to the plant L-domain isoforms while the remaining 258 fungal *CLO/PXG* genes encode proteins analogous to plant H-domain isoforms.

### Functional domains and secondary structures of fungal CLO/PXG proteins

All of the analysed fungal CLO/PXG protein sequences had a similar cluster of functional domains with broadly similar secondary structures when compared to their orthologs in the Viridiplantae (Figs 2, 3 & 4, Additional files 11, 12, 13, 14 and Additional file 15: Figure S2, Rahman et al., 2018). As shown in Fig. 2a, which compares sequences from 40 representative fungal species, the five major conserved domains that are shared with plant CLO/PXG proteins are as follows: N-terminal H-domain, Ca^2+^ binding EF hand, lipid-binding domain, heme-binding and kinase domain, C-terminal phosphorylation domain. In terms of the MEME-predicted motifs described above, the N-terminal H-domain corresponds to motif 6, the Ca^2+^ binding EF hand corresponds to motif 1, the lipid-binding domain corresponds to motif 2, the heme-binding and kinase domain corresponds to motif 3, and the C-terminal phosphorylation domain corresponds to motif 5. Motif 4 is a relatively weak feature and, although there is some conservation of a few invariant residues in this part of the protein, they do not have any obvious functional attributes. Note that, as discussed above, the H-domain is not always present but the other four key domains are well conserved in all fungal species with many invariant or near-invariant residues in all cases. In Fig. 2b, the alignments of 23 sequences from 17 different non-Dikarya species are shown. As expected, these sequences show considerably more diversity because they are taken from species in six different phyla that probably diverged as much as 500 to 1000 Mya (Berbee & Taylor, 2010). The results in Additional files 11, 12, 13, 14 and 15: Figure S2A-E show more detailed alignments for all the Basidiomycota and Ascomycota and for the selected key genera, *Aspergillus, Penicillium, Fusarium* and *Colletotrichum*. In all cases there are relatively minor inter- and intra- genera amino acid differences that are nearly always conservative substitutions but the key functional motifs are very highly conserved.

One structural feature of fungal CLO/PXG proteins that contrasts with those from plants is the number of predicted transmembrane (TM) domains. In Fig. 3, it can be seen that all 40 of the selected fungal sequences contain a predicted TM domain immediately adjacent to the Ca^2+^ binding EF hand motif, but that 19 of these sequences also contained a second TM domain located towards the C-terminus. Results for all 344 fungal sequences are shown in Additional file 16: Figure S3A and results for *Penicillium*, *Fusarium* and *Colletotrichum* spp. are shown in Additional file 17: Figure S3B. These results confirm the general finding that fungal CLO/PXG proteins contain one near-invariant TM domain near the N-terminal and that a subset of the proteins contains a second TM domain near the C-terminus. In contrast, in plants we previously found that out of > 1310 sequences, all but five contained just a single TM domain adjacent to the Ca^2+^ binding EF hand motif [4]. Note that although the data in Fig. 4 were generated using Geneious, we also used several additional TM prediction algorithms with very similar results (see Methods). This indicates that at least some of the fungal CLO/PXG proteins may have different membrane orientations compared to their plant orthologs.

### Gene structures of fungal *CLO/PXG*s

The summary gene structures of *CLO/PXG*s from the 40 selected fungal genomes are shown in Fig. 4 and additional details are presented in Additional file 6: Table S6A. The full lists of gene structures in all available fungal *CLO/PXG*s genes are shown in Additional files 5, 6, 7, 8, and 9: Table S4B - S4D and diagrams of all fungal intron/exon locations are given in Additional files 20, 21, 22, 23, 24, 25, 26 and 27: Figure S4A-H. The fungal *CLO/PXG* genes are highly variable in terms of both the overall numbers and positions of their introns and exons, with intron numbers ranging from zero to 11 across the fungi. In comparison with the recently analysed *CLO/PXG*s from the Viridiplantae [4], which are also quite variable, the fungal genes show even higher levels of variability. This heterogeneity of gene organisation is consistent with the fungal *CLO/PXG*s being members of an ancient gene family that has diverged considerably at the genome level while maintaining relatively conserved protein domain architectures and, possibly, biological functions.

A rather unusual feature of the fungal *CLO/PXG* gene sequences compared to those of plants is the presence of significant numbers of sequences that contain just a single exon and are therefore classified as intronless or single-exon genes [79]. For example, in the Basidiomycota, three out of 89 (3.4%) *CLO/PXG* sequences are intronless, while a further 8 (9%) contain only one intron (Additional file 20 and 21: Figure S4A-B). Meanwhile, in the best studied fungal group, the Ascomycota, 42 out of 231 (18%) genes are intronless while a further 31 (13%) contain only one intron (Additional files 22, 23, 24, 25 and 26: Figure S4C-G). In all of the *CLO/PXG* sequences of non-Dikarya genes, two out of 24 sequences (8%) are intronless while a further three (12%) contain only one intron (Additional file 27: Figure S4H). These results contrast sharply with our recent analysis of 67 *CLO/PXG* gene sequences from plants where all of the genes contained at least two introns and the majority contained between five and six introns [4]. While intronless genes are frequently found in single-celled eukaryotes they are much less common in multicellular plants animals and fungi. Hence, in the Metazoa, between 3 and 17% of genes are intronless in deuterostomes (including 3% in humans and 8% in mice). In contrast, about 20% of genes are intronless in well-studied plant species such as rice and Arabidopsis [80].

In the fungi, intronless genes are rare and there is evidence that the ancestral fungal (and animal) genomes were especially intron-rich with average intron densities in genes from major fungal groups such as Ascomycetes and Basidiomycetes reported to be among the highest among all of the eukaryote groups [81]. Extant fungal genomes are very small in comparison with those of most extant plant and animal genomes, with typical sizes averaging below 45 Mb [76]. This may have been due to a considerable reduction in genome size in fungi following their divergence from other Opisthokonts. At the level of individual genes, the subsequent evolution of the fungi has been characterised by a mixed picture with the maintenance of intron-rich genomes in some lineages but a sharp reduction in intron density in others. Indeed, some Ascomycete genomes are reportedly almost completely intronless, which is very unusual for such advanced eukaryotic species and may be correlated with the overall reduction in genome size in fungi [81]. The data from Fig. 4 and Additional file 4: Table S4 and Additional files 20, 21, 22, 23, 24, 25, 26 and 27: Figure S4A-H show that there are many intronless *CLO/PXG* genes in the well-studied Ascomycete species, and somewhat fewer in the Basidiomycetes. Interestingly, however, two of the *CLO/PXG* genes from the basal species, *Mitosporidium daphniae* and *Paramicrosporidium saccamoebae* are also intronless. Since it is thought that intron loss is normally a derived character in fungal genomes, and occurs more frequently in the more recently evolved Ascomycetes [81], it is interesting that even highly basal clades might be subject to similar processes.

### Functional analysis of CLO/PXG-like proteins in fungi

#### The *A. flavus* CLO/PXG has specific functions in sporulation and aflatoxin production

There are several physiological roles of CLO/PXG proteins that are specific to fungal development. For example, in the common pathogenic fungus, *Aspergillus flavus*, they have been shown to play important roles in various aspects of aflatoxin storage, transport and secretion that have only been recognised very recently [2, 3] where it was reported that the silencing of *A. flavus* caleosin *AfPXG or AflCLO*, the first characterized fungal caleosin with an experimentally proven PXG activity [82], reduced fungal aflatoxicogenicity and its capacity to infect maize [3]. A similar role for *A. parasiticus* caleosin gene was also suggested [83].

**Fig. 5:**
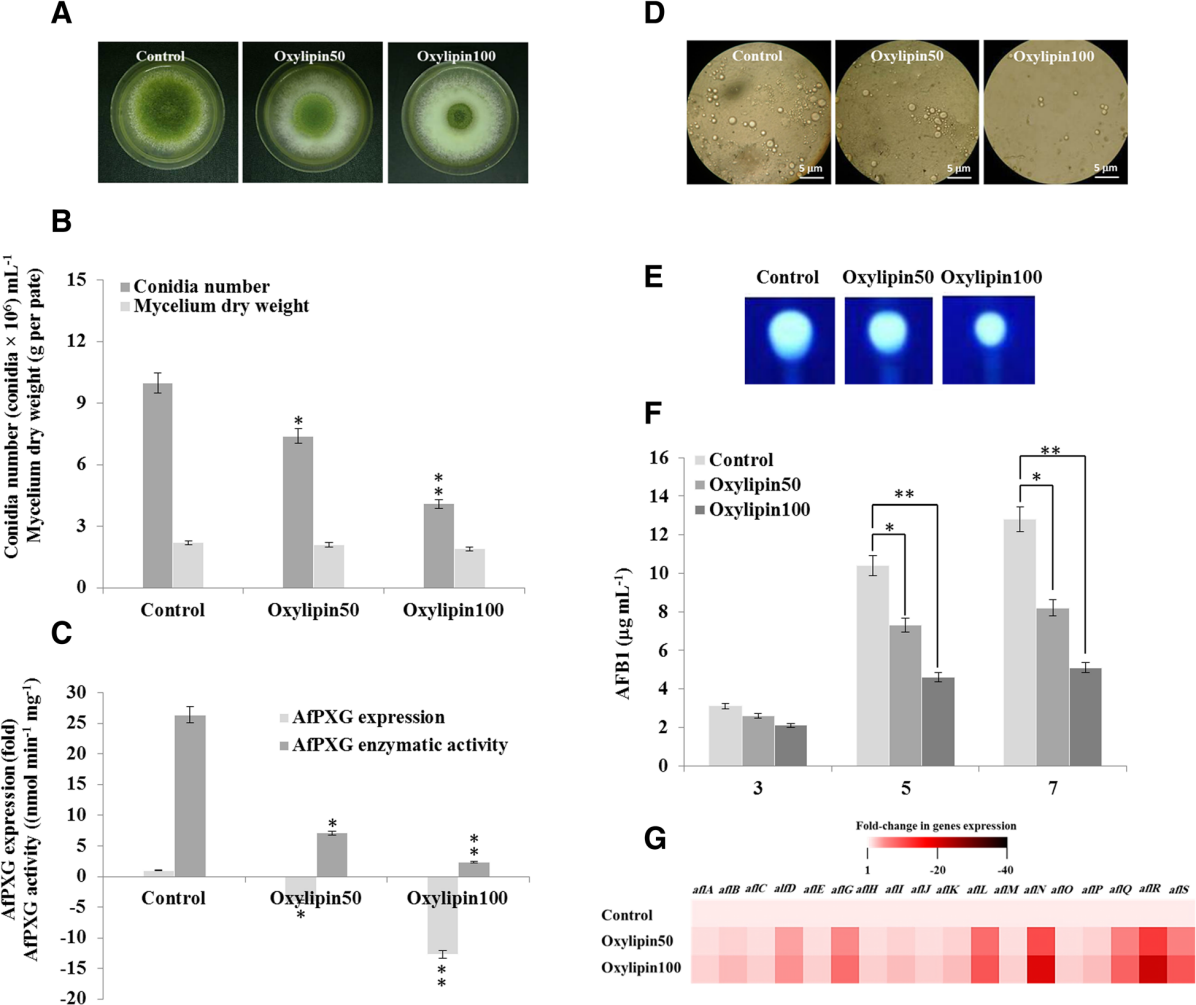
Roles of oxylipins in mediating downregulation of *AfPXG* gene expression and decreased aflatoxicogenicity in *A. flavus*. **a** 7-day old fungal growth on PDA-plates in the presence or absence of exogenous oxylipins at concentrations of 50 and 100 μM, referred to as to oxylipin50 and oxylipin100, respectively. **b** Measurements of conidia number and fungal mycelium dry weight for each treatment compared with controls. **c** Transcript levels of *AfPXG* genes, evaluated by RT-qPCR, and peroxygenase activity of AfPXG, measured by the hydroxylation of aniline at 310 nm. **d** Micrographs of LDs viewed at 40× magnification immediately after preparation. Bar represents 5 μm. **e** Sections of TLC-plate showing the blue-fluorescent spot under UV corresponding to AFB1 extracted from oxylipin-treated fungi compared to a control. **f** Quantitative data for AFB1 production estimated by UV-detector HPLC. **g** Relative quantification RQ _=_ 2^(−∆∆*C*T)^ of AF-biosynthesis cluster genes in oxylipin-treated fungi compared to controls. The colour scale (white-red-black) indicates relative changes of transcripts of 1, − 20 and − 40 fold, respectively where the expression level for each gene in controls was defined as 1

The data presented in Fig. 5 show that oxylipins decrease the aflatoxicogenicity of *A. flavus* in vitro, probably via the down-regulation of the fungal *CLO/PXG* gene, *AfPXG.* As shown in Fig. 5 a, in vitro treatment of *A. flavus* with oxylipins extracted from maize seedlings significantly affects fungal development. While the fungal mycelium weight was not affected as a function of oxylipin concentration, the fungi sporulated less actively under such treatment. This was found in all of the oxylipin-treated fungi with the highest dose (100 μM) producing about 6 × 10^6^ spores per mL fewer than controls (Fig. 5b). In parallel, the transcript level of the major *A. flavus CLO/PXG* gene, *AfPXG*, was reduced by about 12.7-fold after exposure to oxylipins at 100 μM. In parallel, the peroxygenase activity of AfPXG, as evaluated by hydroxylation of aniline, was decreased by the same order of magnitude (Fig. 5c). Moreover, the oxylipin-induced reduction of AfPXG activity was synchronized with a significant reduction in the accumulation LDs in fungal cells, as shown in the light micrographs in Fig. 5d.

We recently reported that both the enzymatic activity of AfPXG and the accumulation of LDs are important for biosynthesis and exporting of aflatoxins (Hanano et al., 2018a). To investigate this further we measured the effects of exogenous oxylipins on fungal aflatoxicogenicity. Images of a sectioned-TLC plate (Fig. 5e) show that the intensity of UV-fluorescent spots corresponding to aflatoxin B1 (AFB1) was reduced as a function of treatments with 50 or 100 μM of oxylipins. This was quantitatively confirmed by HPLC-analysis, where the concentration of AFB1was reduced of about 35 and 60% after oxylipins administration at 50 or 100 μM, respectively, compared to controls (Fig. 5f). The severe reduction in AFB1 levels caused by exogenous oxylipins was related to significant declines in the transcript levels of some key genes of AFB1 biosynthesis, e.g., *alfD*, *alfG*, *alfL*, *alfN*, *alfQ*, *alfR* and *alfS*, where their transcripts were lowered by about 5 to 23-fold compared to control as shown in Fig. 5g. Together, these data suggest that plant oxylipins exert a negative control over the development and aflatoxicogenicity of *A. flavus*, at least in vitro, and that this may be due to down-regulation of the fungal caleosin/peroxygenase, AfPXG.

The link between fungal sporulation and aflatoxicogenicity shown in Fig. 5 is interesting in view of the proven biological connection between conidiation and AF production [84]. Moreover, inhibitory effects of plant oxylipins on fungal growth and aggressivity have also been reported [85, 86]. Our results also showed that this defect in aggressivity was related to the down-regulation of AfPXG at transcript and protein levels. In this context, the genetic involvement of AfPXG in controlling *A. flavus* development and aflatoxicogenicity has been recently reported [3] suggesting that *AfPXG* should be regarded as another gene that is targeted by plant defence mechanisms.

### Putative *CLO/PXG* genes/proteins in a three non-fungal opisthokonts

**Fig. 6:**
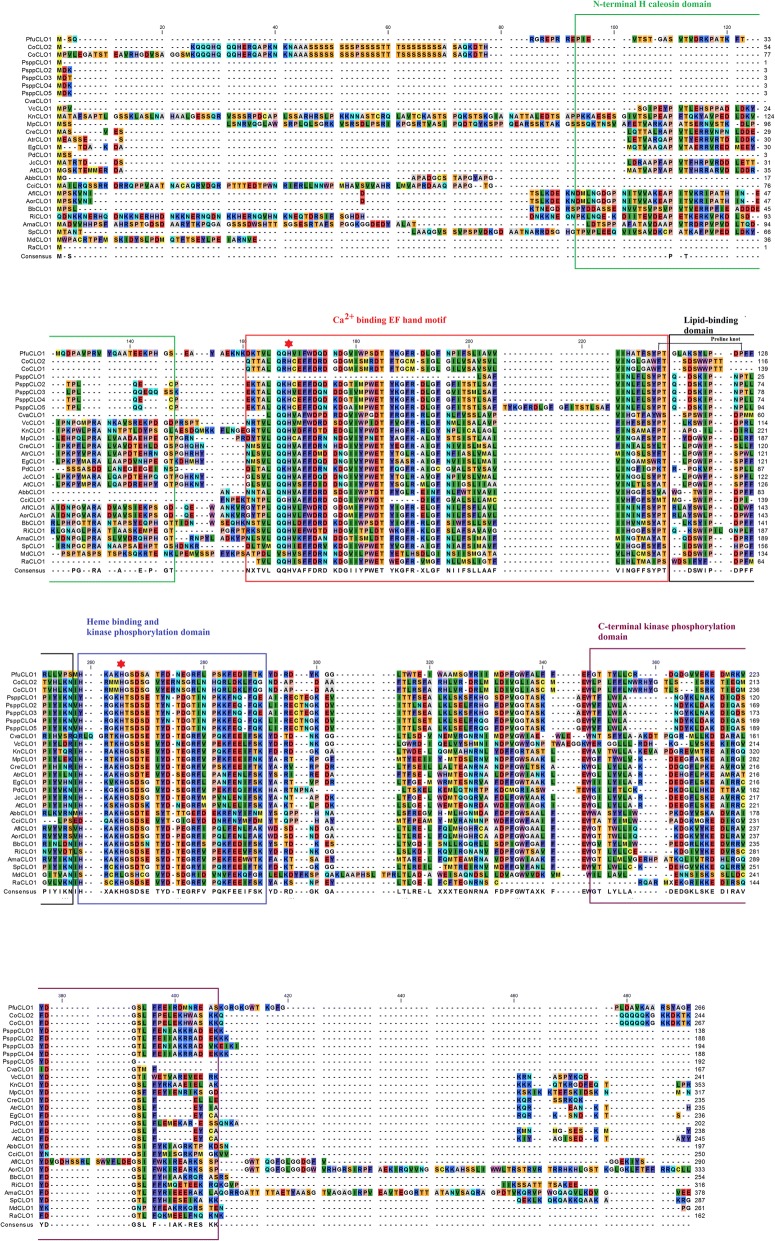
Alignment of CLO/PXG sequences from three non-fungal opisthokont species with ten selected fungal and ten plant sequences. The three non-fungal opisthokont are: *Planoprotostelium fungivorum* (PfuCLO1), *Capsaspora owczarzaki* (CoCLO1 & 2), and *Panagrolaimus spp* (PsppCLO1–5). The ten Viridiplantae species are: *Chlorella variabilis* (CvaCLO1), *Volvox carteri* (VcCLO1), *Klebsormidium nitens*(KnCLO1), *Marchantia polymorpha*(MpCLO1), *Cycus revolute* (CreCLO1), *Amborella trichopoda* (AtrCLO1), *Elaeis guineensis* (EgCLO1), *Phoenix dactylifera* (PdCLO1), *Jatropha curcas* (JcCLO1) and *Arabidopsis thaliana* (AtCLO1). The ten fungal species are: *Agaricus bisporus* (AbbCLO1), *Coprinopsis cinerea* (CciCLO1), *Aspergillus flavus* (AflCLO1), *Aspergillus oryzae* (AorCLO1), *Beauveria bassiana* (BbCLO1), *Rhizophagus irregularis* (RiCLO1), *Allomyces macrogynus* (AmaCLO1), *Spizellomyces punctatus (*SpCLO1), *Mitosporidium daphnia* (MdCLO1) and *Rozella allomycis (*RaCLO1)

The data presented above are consistent with the occurrence of *CLO/PXG* genes in the ancestor of the monophyletic fungal clade and the subsequent loss of these genes in about 70% of modern fungal genomes, but their retention in the other 30%. This begs the question as to whether *CLO/PXG* genes were/are also present in other lineages of the Opisthokonta. Sequence data are now available for many hundreds of Opisthokont genomes but we were only able to find putative *CLO/PXG* genes in three of these genomes. We have recently reported on two of these cases, namely the basal Opisthokont, *Capsaspora owczarzaki* and the Metazoan (nematode) genus, *Panagrolaimus spp* [4] and we here describe a third *CLO/PXG* gene from Amoebozoan, *Planoprotostelium fungivorum*. An alignment of CLO/PXG sequences from these three non-fungal opisthokont species with ten selected fungal and ten plant sequences is presented in Fig. 6.

*C*. *owczarzaki*, is a Holozoan amoeboid symbiont of the pulmonate snail, *Biomphalaria glabrata*, and is a member of a lineage that is more closely related to the Metazoa than to the Fungi [87]. Given the important roles of CLO/PXGs in LD accumulation in both plants and fungi [3], it is of interest that during specific phases of its life cycle, *C*. *owczarzaki* cells can both accumulate and extrude LDs. Although there are not very high levels of sequence similarity when the two putative *C*. *owczarzaki CLO/PXG* sequences are subjected to open BLAST searches, the retrieved fungal sequences are more similar than those of plants. This is consistent with *C*. *owczarzaki CLO/PXG* sequences being more closely related to fungi than plants. However, whether this is due to HGT or simply because the *C*. *owczarzaki CLO/PXG* genes retained more plant like sequences after the post-LECA divergence, remains an open question.

The situation is less clear for the gene from the nematode genus, *Panagrolaimus spp*, where BLAST searches with the five putative *CLO/PXG* sequences all showed high similarity scores with both plants and fungal *CLO/PXG*s. Again, it is not possible to rule out the possibility that these genes were secondarily acquired by the nematode via HGT, although if this were the case it is not clear whether the donor was a plant or a fungus. As for the possible roles of CLO/PXGs in this nematode (which is apparently unique among Metazoa in harbouring such genes) the putative *CLO/PXG* genes were only found in parthenogenetic species in the genus and are functionally linked to cryptobiosis and especially to desiccation tolerance [88]. This is interesting because algal caleosins are highly upregulated following salt stress and this may have played a role in the transition of more complex plants from aquatic to terrestrial environments with the concomitant requirement for improved tolerance to desiccation [4].

The third non-fungal opisthokont genome with a putative *CLO/PXG* gene is the Amoebozoan, *Planoprotostelium fungivorum*, which is an exclusively mycophagous amoeba that predates upon a range of fungi that include *Candida* and *Aspergillus* spp. In this case open BLAST-P searches give the highest sequence similarity with CLO/PXGs from the Pezizomycotina, and especially Eurotiomycetes.

### Comparative phylogenetics of *CLO/PXG* genes in fungi and plants

**Fig. 7:**
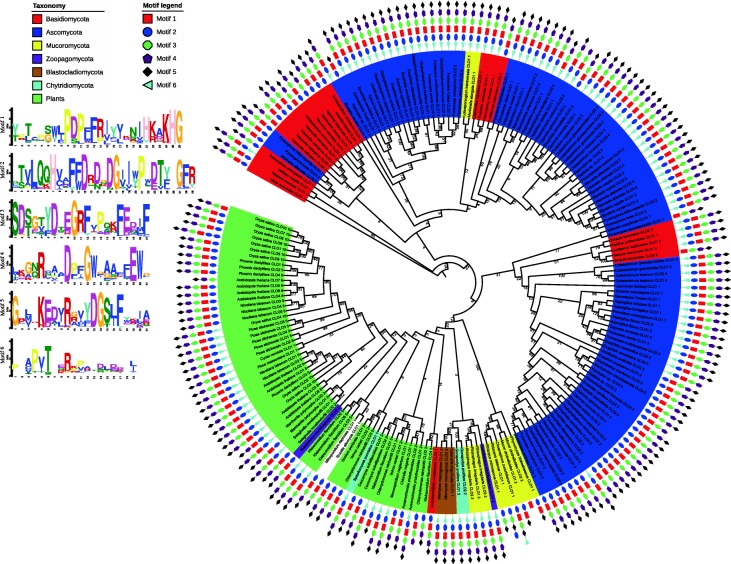
Representative Maximum Likelihood phylogeny for 199 plant and fungal CLO/PXG proteins. The optimum model of protein substitution was found to be LG + G. Bootstrap resampling (100 iterations) was undertaken and is shown on internal nodes. There are several strongly supported clades but support values inferring sister group relationships between these are extremely low. Species names are coloured relative to their taxonomy. The presence of MEME predicted motifs are shown for individual proteins

The phylogenetic history of all 462 plant and fungal CLO/PXG proteins was investigated using the Maximum Likelihood framework implemented in PhyML with the optimum evolutionary model determined by ProtTest. Based on this original phylogeny (Additional file 28: Figure S5), a second representative phylogeny consisting of 199 proteins was also reconstructed (Fig. 7). Motifs common to all proteins were determined using MEME and represented on both phylogenies (Fig. 7 & Additional file 28: Figure S5). Overall, six unique motifs were located and most proteins contained at least 5 of these (Fig. 7 & Additional file 28: Figure S5).

Our phylogenies indicate that there is poor phylogenetic signal in our dataset. This is evident as bootstrap supports associated with internal nodes linking individual strongly supported clades are extremely low (Fig. 7 & Additional file 28: Figure S5). This lack of signal is most likely the result of the highly conserved nature of the CLO/PXG domain. While the phylogenetic signal in our dataset is low it is worth noting that it is better than random. To demonstrate this we constructed constrained trees that grouped CLO/PXG proteins together based on their associated phyla, we also generated 100 random trees. According to the approximately unbiased test our representative phylogeny is better than all random or constrained trees (*P* < 0.003).

Our representative phylogeny shows that all the plant CLO/PXG proteins are grouped together in a single polyphyletic clade along with a number of basal fungal groups namely the *incertae sedis* species *Mitosporidium daphnia* and *Rozella allomycis* along with the Zoopagomycota species *Basidiobolus meristosporus*. Next to this clade we find species belonging to the Blastocladiomycota (*Allomyces macrogynus* and *Catenaria anguillulae*), Chytridmycota (*Spizellomyces punctatus* and *Gonapodya prolifera*) and Mucoromycota phyla (Fig. 7). As noted above the branch supports for these groupings are extremely poor and in our view are not indicative of strong sister group relationships. Similarly the phylogeny with all 462 proteins present also displays very poor bootstrap support values (Additional file 29: Figure S6).

To illustrate the degree of phylogenetic disagreement within the dataset, we reconstructed a neighbour-joining network of phylogenetic splits within the representative dataset alignment (Additional file 29: Figure S6). The network is highly congruent with the representative phylogeny (Fig.7). Strongly supported clades found in the phylogeny can also be clearly seen in the network. However, there is a large degree of conflict regarding the sister group relationships amongst these clades as illustrated by the large number of alternative splits between clades (Additional file 29: Figure S6). Based, on both our phylogenetic and network analyses we suggest it is not possible to confidently infer the true phylogenetic relationships amongst these proteins.

A recent in silico analysis of the Mucoromycota species, *Rhizophagus irregularis* has suggested that this arbuscular mycorrhizal fungus has acquired 19 genes via horizontal gene transfer from plants and bacteria [69]. One of these genes is reported to be a CLO/PXG gene from a plant donor. Our phylogenetic analysis fails to confirm this finding. Firstly, other Mucoromycota species are grouped beside the *R. irregularis* CLO/PXG genes (Fig. 7). Therefore, if HGT did occur and *R. irregularis* contains a plant CLO/PXG ortholog, it was not a recent transfer into *R. irregularis* alone (Fig. 7 & Additional file 28: Figure S5). Secondly, the bootstrap supports and the network analysis grouping the basal fungi with the plants is very low (as described above). To infer that a HGT event has occurred, would mean that *R. irregularis* and indeed the ancestor of other basal fungi lost the fungal CLO/PXG ortholog and acquired the plant copy instead, meanwhile Dikarya species retained a fungal ortholog. This scenario is most un-parsimonious, what is more likely is that the CLO/PXG gene was present in the last eukaryote common ancestor (LECA) and was subsequently lost in all lineages except for the fungal and plant kingdoms. The presence of CLO/PXG orthologs in all plant genomes sequenced to date would suggest that it is an important core housekeeping gene while the somewhat more patchy phyletic distribution amongst fungal species (even sister species) suggests that it is an accessory gene that may be beneficial to have in particular environments or niches.

## Discussion

Three general observations can be made from our analysis of the distribution of *CLO/PXG*-like sequences in fungal genomes. ***First***, this gene family is present in all the major fungal groups from the most primitive to the most recently evolved. Interestingly, *CLO/PXG*-like sequences are present in the two basal clades, Microsporidia and Cryptomycota, which have only very recently (and, to date, not unanimously) been accepted as likely members of the Fungi [89–94]. ***Second***, the CLO/PXG-like protein sequences are generally well conserved and their major canonical motifs (see below) are strikingly similar to orthologs that are highly abundant in the Viridiplantae [4]. ***Third***, and unlike most of the Viridiplantae groups, *CLO/PXG*-like sequences are absent from a large proportion (about 70%) of currently sequenced fungal genomes. In a few cases, such as the Orbiliomycetes and Lecanoromycetes, current information indicates that the genes are absent from an entire class of fungi. However, in these classes only a few species have so far been sequenced so it might be the case that *CLO/PXG*-like genes are in fact present in some members of all fungal classes. In many other cases, such as in the important genera, *Aspergillus, Penicillium* and *Colletotrichum*, the genes are present in one or more members of each genus but are absent from close relatives in the same genus. These findings imply that in the Fungi as a whole *CLO/PXG* genes were probably originally present in all of the early diverging taxa but they have frequently been lost during subsequent evolution, albeit being retained in almost one third of fungal species.

In terms of their subcellular orientation, almost all of the fungal CLO/PXG proteins were predicted to have an ‘extracellular’ facing N-terminal domain containing the Ca^2+^ binding EF hand motif (Additional file 18: Figure S3C). In proteins with a single TM domain this means that they would have a large cytoplasmic domain containing several highly conserved regions including kinase sites while in proteins with two TM domains there is a smaller cytoplasmic domain and two ‘extracellular’ facing domains (Additional file 19: Figure S3D). In terms of ER-binding proteins the ‘extracellular’ domain is equivalent to the lumen compartment while for plasmalemma proteins it really is extracellular. Therefore the Ca^2+^ binding and heme-coordinating regions of fungal CLO/PXG proteins are probably located either in the ER lumen or outside the cell membrane while there are several kinase sites on the cytoplasmic portions of these proteins. These characteristics are consistent with roles for CLO/PXGs in processes such as signalling and the recognition of external agents, e.g. as part of responses to environmental stress and as components of the pathogenesis process (see also discussion below).

### General overview of CLO/PXG functions in fungi

The finding here that *CLO/PXG* genes are present in about 30% of species in the major fungal groups begs the question: what is the function(s) of *CLO/PXG* genes in Fungi and what is the difference between species that have lost them versus those that have retained them, possibly for as much as one billion years? We describe above new evidence that we have presented for some specific functions of *CLO/PXG* genes that are related to processes involving oxylipin signalling, LD metabolism, and aflatoxicogenicity. However, it is also important to look more broadly at other possible functions of these genes, especially in the light of their apparent loss in so many fungal groups. In this regard, we were unable to discern any systematic differences between those fungal species that lack *CLO/PXG* genes versus those that have retained them. For example, when the economically important genera, Aspergillus (where 30/54 genomes contain *CLO/PXG* genes), Penicillium (7 out of 28 genomes) and Colletotrichum (13 out of 20 genomes), are examined in more detail (see Additional file 2: Table S2) it can be seen that some highly pathogenic species contain *CLO/PXG* genes while other closely related and similarly pathogenic species lack these genes. To date, there has been a relatively limited number of studies on the function of CLO/PXG proteins in fungi and those studies that have been carried out have been restricted to Dikarya species (Table 2).

**Table 2:** CLO/PXG functions in fungi

Higher taxa		Species	Gene/protein designation	Protein location	Biological function(s)	Reference(s)
Glomeromycota		*Rhizophagus irregularis*				
Ascomycota	Pezizmycotina	*Aspergillus flavus*	CLO/PXG	LDs	Lipid storage, aflatoxin storage, transport and secretion	Hanano et al., 2015 [2]; 2018 [3]
		*Beauveria (Cordyceps) bassiana*	Caleosin	LDs	Lipid storage/mobilisation, spore dispersal, virulence, cuticle penetration	Cho et al., 2006 [107]; Fan et al. 2015 [5]; Ortiz-Urquiza et al. 2016 [6]; Chen et al., 2017; Keyhani, 2017 [108]; Chen et al., 2018 [106]
		*Metarhizium robertsii* *Metarhizium anisopliae*			Lipid metabolism, appressorial turgor pressure, virulence, host invasion, N starvation	Gao et al., 2013 [7]; Wang et al., 2007 [8]; Chen et al., 2017
		*Blumeria graminis*	Caleosin	Conidia	Conidia development, LD formation	Zeng et al., 2017 [9]
	Taphrinomycotina					
	*Saccharomycotina*	*Saccharomyces cerevisiae*	*Caleosin*	*LDs*	*CLO gene absent but heterologous plant expressed and protein found on LDs*	Froissard et al., 2009 [47]
Basidiomycota	Agaricomycotina	*Rhodosporidium toruloides*				Zhu et al., 2015 [11]
	Ustilagomycotina					
	Pucciniomycotina	*Puccinia graminis*	Peroxygenase		Pathogenesis response, oxylipin metabolism	Rutter et al., 201 [10]
Microsporidia – *incertae sedis*		*Mitosporidium daphnia,*				
Cryptomycota/Rozellomycota		*Rozella allomycis,* *Paramicrosporidium saccamoebae*				
Blastocladiomycota		*Allomyces macrogymus,* *Catenaria anguillulae*				
Chytridiomycota		*Spizellomyces punctatus* *Gonapodya prolifera*				
Neocallimastigomycota		*Neocallimastix californiae*				
Zoopagomycota		*Basidiobolus meristosporus*				
Mucoromycota		*Mucor circinelloides,* *Rhizopus delemar/oryzae;* *R. microsporus* *Mucor circinelloides; Parasitella parasitica, Phycomyces blakesleeanus, Lobosporangium transversale* *Choanephora cucurbitarum* *Mortierella elongata*				

*CLO/PXG* gene presence, expression patterns and protein functions in fungal taxa

Gene knockout and biochemical studies have confirmed that, as in land plants and algae [4], fungal CLO/PXG proteins have multifunctional roles that include a structural role in lipid droplet and aflatoxin formation, storage and mobilisation [2, 3, 7] and enzymatic roles as peroxygenases in stress-related physiological responses including oxylipin metabolism [95]. Many species of fungi form intimate associations with plants and algae that range from benign symbioses such as in mycorrhizae and some lichens to a spectrum of pathogenic interactions from relatively well tolerated long-term associations to rapidly acting, virulent and often lethal diseases. However, while both the plant and fungal partners in mycorrhizal associations might express their respective *CLO/PXG* genes (which appear to be phylogenetically distinct) as part of this process, there is evidence that these genes are essential for a successful mycorrhizal development.

Oxylipin pathways in both plant and fungal partners can play crucial roles in mediating the crosstalk between many plant hosts and their fungal pathogens, which can determine the eventual outcome of the pathogenesis process [23, 96–98]. In several cases in plants it has been reported that CLO/PXG peroxygenase activity plays a direct role in plant responses to fungal pathogens. Examples include the response of wheat plants to infection by the pathogens, *Puccinia graminis* and *P. stritiformis*, where *CLO/PXG* genes have been shown to be involved in the host-pathogen co-expression network during infection of wheat plants [10, 97, 98]. Plant lipid droplets (LDs) are both one of the major subcellular sites of CLO/PXG accumulation and also contain oxylipins that are involved in anti-fungal defences [31, 99–101]. In line with this, we have demonstrated that the *A. flavus* AfPXG has peroxygenase activity and mediates fungal development and aflatoxin production [82].

A more recent report has shown that the *AfPXG*-deficient line resulted in a fungal phenotype characterized with a severe decrease in mycelium growth, failure in sporulation and a reduced level of aflatoxin. Inversely, the line that overexpressed *AfPXG*, with the reporter gene *Gfp* exhibited an elevated numbers of stable lipid droplets (LDs) plus enhanced aflatoxin levels [3]. In addition, the transcriptomic profile of *Blumeria graminis* f. sp. *tritici* showed that a Ca^+ 2^-binding protein is involved in fungal conidiation [9]. Also, a similar situation was observed in *Metarhizium robertsii* [7]. In addition to their developmental impacts, CLO/PXGs can act as cellular protective agents against toxins and highly hydrophobic contaminants via their roles in stabilizing the structure of toxin-sequestrating LDs [41, 102, 103]. It has also been observed that plant CLOs/PXGs affect development and the pathogenicity of fungal pathogens via the anti-fungal compounds generated by lipid-metabolizing activities [10, 20, 104] .

CLO/PXGs have also been implicated in fungal pathogenicity in insect hosts. In the entomopathogenic species, *Beauveria (Cordyceps) bassiana*, caleosins are involved in several aspects of both reproduction and pathogenesis. This was based on the identification of a single LD-surface caleosin-like protein with roles in lipid storage and fungal infection [5]. Although its peroxygenase activity has yet to be experimentally verified, this caleosin, referred as to BbCLO1, possesses all structural and functional features typically found in plant CLOs. It was shown that the Δ*BbCLO1* mutant produced more compact assemblages of conidia, displayed a reduced spore dispersal phenotype and a decreased virulence against insects [5]. It was found that the contribution of this caleosin to virulence was greater during pre-penetration/penetration events when the fungus was grown on oleic acid suggesting critical roles for caleosin-mediated lipid mobilization during the initial phases of fungal infection [6]. With a less direct connection to CLOs, genetic alterations that reduce triacylglycerol (TAG) biosynthesis or formation of LDs and their associated proteins can seriously affect insect invasion by *Metarhizium robertsii* and *M. anisopliae* [7, 8, 105]. Moreover, *CLO/PXG*-gene knockouts are compromised (but not necessarily fatally) in their ability to disperse spores efficiently. These deletion mutants are also less virulent than wild-type strains with reduced ability for penetration into the cuticle of their insect hosts and for the acquisition of lipid nutrients from the latter [5, 6, 106–108]. It is also reported that during the infection process on ants, *B. bassiana* is able to metabolise the host cuticular lipids, by means of hydrocarbon and fatty acid oxidation, in a manner that contributes to fungal virulence [109].

To date there have been no reports of functional studies involving *CLO/PXG* genes in non-Dikarya fungal groups. However, given the important roles of lipid droplets in the transport of carbon from host plants to mycorrhizal species [110], it would be useful to study possible roles for CLO/PXG proteins in such fungi. Interestingly, in the only sequenced Glomeromycotina species, *Rhizophagus irregularis*, there are no fewer than three *CLO/PXG*–like genes (Additional file 3: Table S3). As noted above, entomopathogenic fungi use specialized structures, termed appressoria, to break through the cuticular surfaces of their hosts and similar structures are found in phytopathogenic fungi. It is known that lipid droplets are required for appressorium function in order to maintain the high turgor pressure that is required for virulence [8, 111]. In both the rice blast fungus, *Magnaporthe grisea* [111], and the insect pathogen, *Metarhizium anisopliae* [8], lipid droplets originate in fungal spores and redistribute to the incipient appressorium. Lipid droplets also play roles in colonization and sexual development in other fungi including the wheat pathogen, *Fusarium graminearum* [112], and *Aspergillus nidulans* [113], which is both a soil-dwelling fungus and opportunistic human pathogen.

Interestingly, in the widely studied Saccharomycetes class, only one out of the 48 sequenced species contains *CLO/PXG* genes. Therefore, *CLO/PXG* genes are absent from such important experimental species as brewers’ yeast, *Candida* spp. and *Pichia* spp. but are present in the closely related yeast, *Lipomyces starkeyi* (Additional file 3: Table S3). However, when *CLO/PXG* genes from either fungi or plants are expressed in the normally *CLO/PXG* genes-deficient yeast, *Saccharomyces cerevisiae*, the genes are expressed and active CLO/PXG proteins are produced, leading to increased accumulation of intracellular lipid droplets that harbour these proteins [2, 47]. Another interesting CLO/PXG-containing fungus is the basidiomycetous yeast, *Rhodosporidium toruloides*, which has biotechnological uses as a producer of carotenes and triacylglycerols. Although the genome of this species has yet to be fully sequenced, laboratory studies have shown that *CLO/PXG* genes are highly induced in response to nitrogen starvation and that the resultant CLO/PXG proteins are one of the two major components of the lipid droplet proteome [11]. Since the overexpression of *CLO/PXG* genes in *Saccharomyces cerevisiae* leads to increased triacylglycerol accumulation [2, 47], the manipulation of these genes in other commercially useful lipogenic fungi could be part of a biotechnological approach to increasing lipid production for use in oleochemicals, foods or biofuels. Given our recent demonstration of the close relationship between CLO/PXG proteins, lipid droplet dynamics and aflatoxin biosynthesis, transport and export in *A. flavus* [3], further studies on the wider roles of these proteins are definitely merited.

In terms of the origins and current distribution of CLO/PXG genes in eukaryotes we suggest that these genes were present in the last eukaryote common ancestor (LECA) that pre-dated the split into the major extant clades such as plants, fungi and Metazoa. The present distribution as reported here suggests that CLO/PXG genes were subsequently lost in all eukaryotic lineages except for the fungal and plant kingdoms. The presence of CLO/PXG orthologs in all plant genomes sequenced to date would suggest that these genes have important core housekeeping functions, especially in the land plants. In contrast, the more patchy distribution CLO/PXG genes amongst fungal species, where even inside many genera some species might contain the gene while others do not, suggests that CLO/PXG is more of an accessory gene in the fungi that may be beneficial to have in particular environments or niches.

## Conclusions

In this study we have analysed the *CLO/PXG* gene family in the fungi and other non-plant clades. This complements our recent analysis of the *CLO/PXG* gene family in the Viridiplantae [4]. Whereas *CLO/PXG* genes are ubiquitous in the land plants (Streptophytes), they are only found in about 30% of the sequenced fungal genomes that we were able to recover from public databases. However, *CLO/PXG* genes are present in all of the major fungal taxa, inferring that they have been selectively lost in some fungal lineages over the past one billion years of evolution. *CLO/PXG*-like genes were also present in only three out of the many hundreds of sequenced non-fungal Opisthokont genomes including an Amoebozoan, a Holozoan and a Metazoan, although whether the unusual presence of these genes is the result of HGT remains an open question.

Functional experiments in this study and elsewhere show that fungal CLO/PXGs have similar but not identical roles to those in plants, including stress-related oxylipin signalling, lipid metabolism, reproduction and pathogenesis. While the presence of CLO/PXG orthologs in all plant genomes sequenced to date would suggest that they have core housekeeping functions in plants, the selective loss of *CLO/PXGs* in many fungal genomes suggests more restricted functions in fungi as accessory genes useful in particular environments or niches. We propose that although *CLO/PXG*-like genes were originally present in LECA, they were then lost in the ancestors of non-fungal Opisthokont taxa such as the Metazoa, following divergence of the latter from ancestral Fungi.

## Additional files

Additional file 1: **Table S1.** Details of various bioinformatics tools and packages used in this study, including web-location and licence type (XLSX 10 kb)

Additional file 2: **Table S2.** Lists of fungal *CLO/PXG*-like sequences. A list of the 844 sequenced fungal genomes analysed by BLAST searches for the presence of *CLO/PXG*-like sequences. (XLSX 50 kb)

Additional file 3: **Table S3.** The full list of the 243 sequenced fungal genomes that between them contained 344 *CLO/PXG*-like sequences plus the CLO names assigned in this study, their NCBI/Genbank gene numbers and other information on the characteristics of the encoded proteins. (XLSX 54 kb)

Additional file 4: **Table S4.** List of 40 representative fungal genomes use for motif and structural analyses including the CLO names assigned in this study, their NCBI/Genbank gene numbers and other information on the characteristics of the encoded proteins. (XLSX 16 kb)

Additional file 5: **Table S5.** Predicted transmembrane regions of 40 representative fungal CLO/PXG proteins. (XLSX 10 kb)

Additional file 6: **Table S6A.** Intron/exon numbers in 40 representative fungal *CLO/PXG* genes. (XLSX 18 kb)

Additional file 7: **Table S6B.** Intron/exon numbers in 89 Basidiomycota *CLO/PXG* genes. (XLSX 15 kb)

Additional file 8: **Table S6C.** Intron/exon numbers in 231 Ascomycota *CLO/PXG* genes. (XLSX 24 kb)

Additional file 9: **Table S6D.** Intron/exon numbers in 24 non-Dikarya *CLO/PXG* genes. (XLSX 9 kb)

Additional file 10: **Figure S1.** Motif analysis of all 344 fungal CLO/PXG sequences. The sequences are in the same order and have the same identifying numbers as the detailed list shown in SI Table 2. The colour scheme for the six motifs is the same as that shown in Fig. 1. (PNG 374 kb)

Additional file 11: **Figure S2A.** CLO/PXG protein sequence alignments from all sequences of Basidiomycota. (PNG 3608 kb)

Additional file 12: **Figure S2B.** CLO/PXG protein sequence alignments from all sequences of Ascomycota. (PDF 7080 kb)

Additional file 13: **Figure S2C.** CLO/PXG protein sequence alignments from all sequences of *Aspergillus* spp. (PNG 429 kb)

Additional file 14: **Figure S2D.** CLO/PXG protein sequence alignments from all sequences of *Colletotrichum* spp. (PNG 1487 kb)

Additional file 15: **Figure S2E.** CLO/PXG protein sequence alignments from all sequences of *Fusarium* and *Penicillium* spp. (PNG 1349 kb)

Additional file 16: **Figure S3A.** Predicted transmembrane domain locations in all 344 fungal CLO/PXG proteins. (JPG 21303 kb)

Additional file 17: Figure S3B, Predicted secondary structures of CLO/PXG proteins from Penicillium, Fusarium and Colletotrichum genera. (PDF 869 kb)

Additional file 18: **Figure S3C.** Predicted subcellular orientation of a fungal CLO/PXG protein with one transmembrane domain (*Allomyces macrogynus*). (PNG 66 kb)

Additional file 19: **Figure S3D.** Predicted subcellular orientation of a fungal CLO/PXG protein with two transmembrane domains (*Aspergillus flavus*). (PNG 70 kb)

Additional file 20: **Figure S4A.** Predicted *CLO/PXG*s gene structures of first group of Basidiomycota. (PNG 268 kb)

Additional file 21: **Figure S4B.** Predicted *CLO/PXG*s gene structures of second group of Basidiomycota. (TIF 268 kb)

Additional file 22: **Figure S4C.** Predicted *CLO/PXG*s gene structures of first group of Ascomycota. (PNG 141 kb)

Additional file 23: **Figure S4D.** Predicted *CLO/PXG*s gene structures of second group of Ascomycota. (PNG 170 kb)

Additional file 24: **Figure S4E.** Predicted *CLO/PXG*s gene structures of third group of Ascomycota. (PNG 160 kb)

Additional file 25: **Figure S4F.** Predicted *CLO/PXG*s gene structures of fourth group of Ascomycota. (PNG 172 kb)

Additional file 26: **Figure S4G.** Predicted *CLO/PXG*s gene structures of fifth group of Ascomycota. (PNG 95 kb)

Additional file 27: **Figure S4H.** Predicted *CLO/PXG*s gene structures of non-Dikarya species. (PNG 86 kb)

Additional file 28: **Figure S5.** Maximum Likelihood phylogeny for all 462 analysed plant and fungal CLO/PXG proteins. The optimum model of protein substitution was found to be LG + G. Bootstrap resampling (100 iterations) was undertaken and are shown on internal nodes. There are several strongly supported clades but support values inferring sister group relationships between these are extremely low. Species names are coloured relative to their taxonomy. The presence of MEME predicted motifs are shown for individual proteins. (PDF 5353 kb)

Additional file 29: **Figure S6.** Phylogenetic network reconstructed using the representative 199 plant and fungal CLO/PXG proteins. The neighbour-joining network method was used to infer splits within the alignment. Species names are coloured relative to their taxonomy. Strongly supported monophyletic clades are evident within the network. However relationships between these clades is conflicting as illustrated by many alternative splits at the base of the network. (PDF 556 kb)

Additional file 30: Accession number and links to the analysed data. (XLSX 38 kb)

## Abbreviations

CLO/PXG: Caleosin/peroxygenase
HGT: Horizontal gene transfer
LD: Lipid droplet
LECA: Last eukaryotic common ancestor
MEME: Multiple En for Motif Elicitation
Mya: Million years ago
NCBI: National Center for Biotechnology Information
Pfam: Protein family
UniProt: Universal Protein resource
