# Supplementary figures and images for: Evolutionary, structural and functional analysis of the caleosin/peroxygenase gene family in the Fungi
Source: BMC Genomics. 2018 Dec 28;19:976. doi: 10.1186/s12864-018-5334-1 (PMC6309107; doi:10.1186/s12864-018-5334-1)

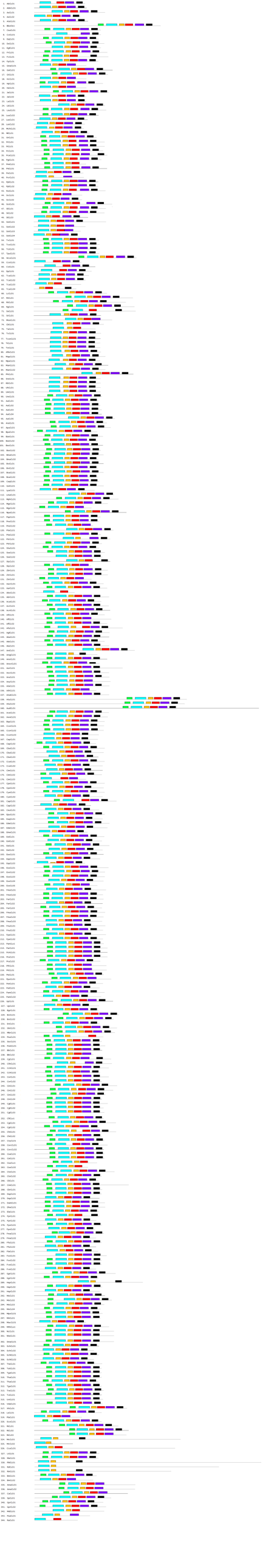

Supplement: Supplementary file 10 — Figure S1. Motif analysis of all 344 fungal CLO/PXG sequences. The sequences are in the same order and have the same identifying numbers as the detailed list shown in SI Table 2. The colour scheme for the six motifs is the same as that shown in Fig. 1. (PNG 374 kb) [file 12864_2018_5334_MOESM10_ESM.png]

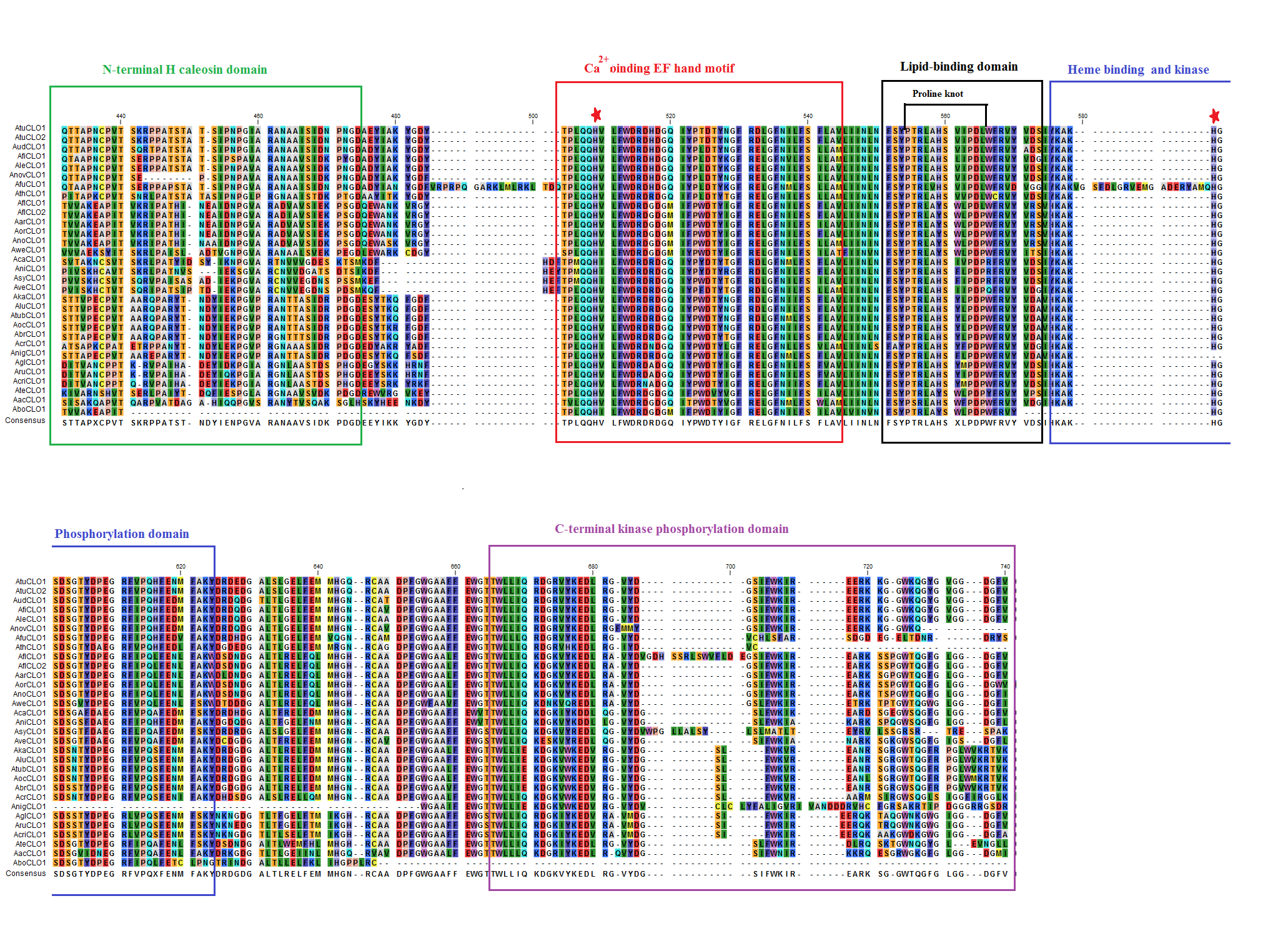

Supplement: Supplementary file 13 — Figure S2C. CLO/PXG protein sequence alignments from all sequences of Aspergillus spp. (PNG 429 kb) [file 12864_2018_5334_MOESM13_ESM.png]

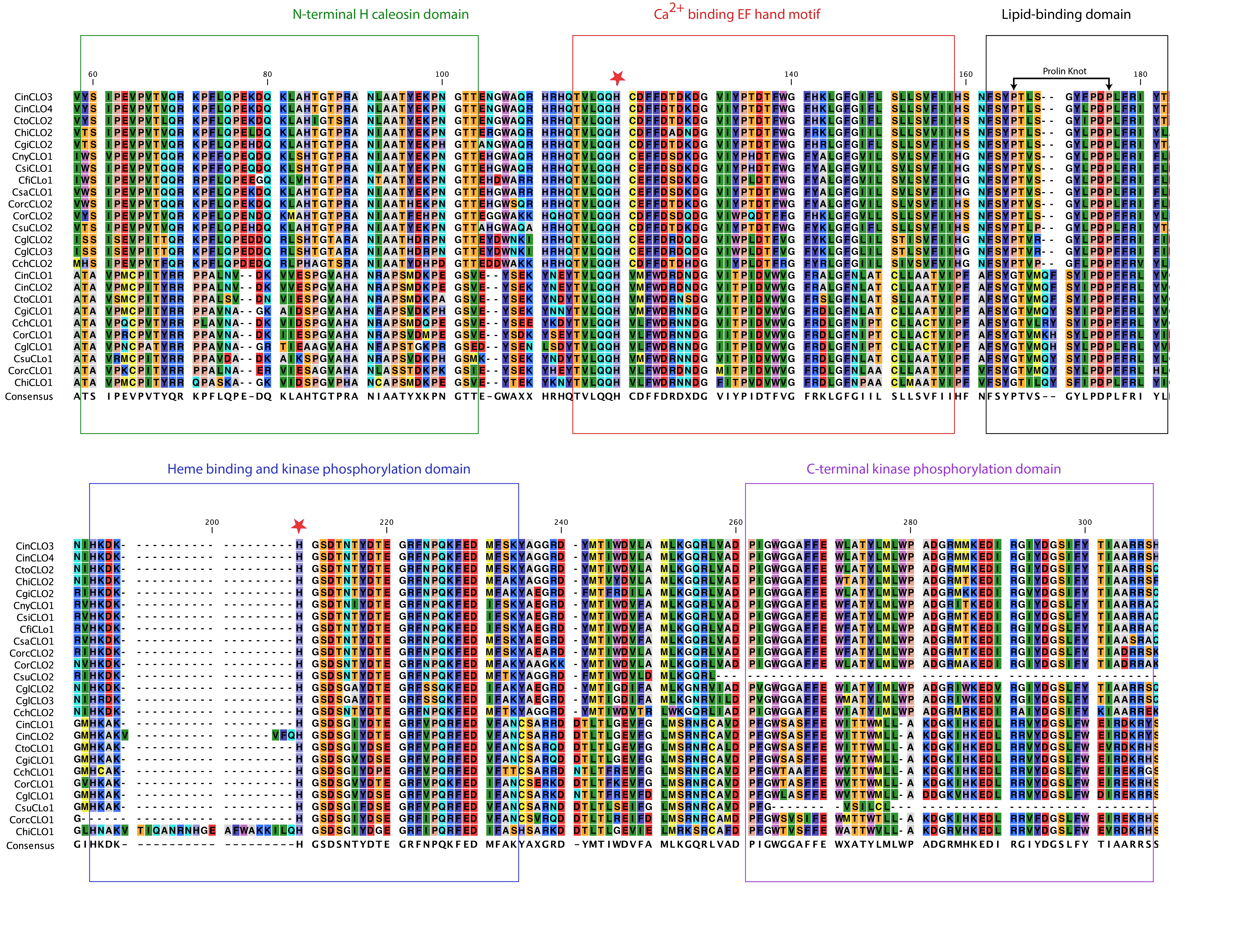

Supplement: Supplementary file 14 — Figure S2D. CLO/PXG protein sequence alignments from all sequences of Colletotrichum spp. (PNG 1487 kb) [file 12864_2018_5334_MOESM14_ESM.png]

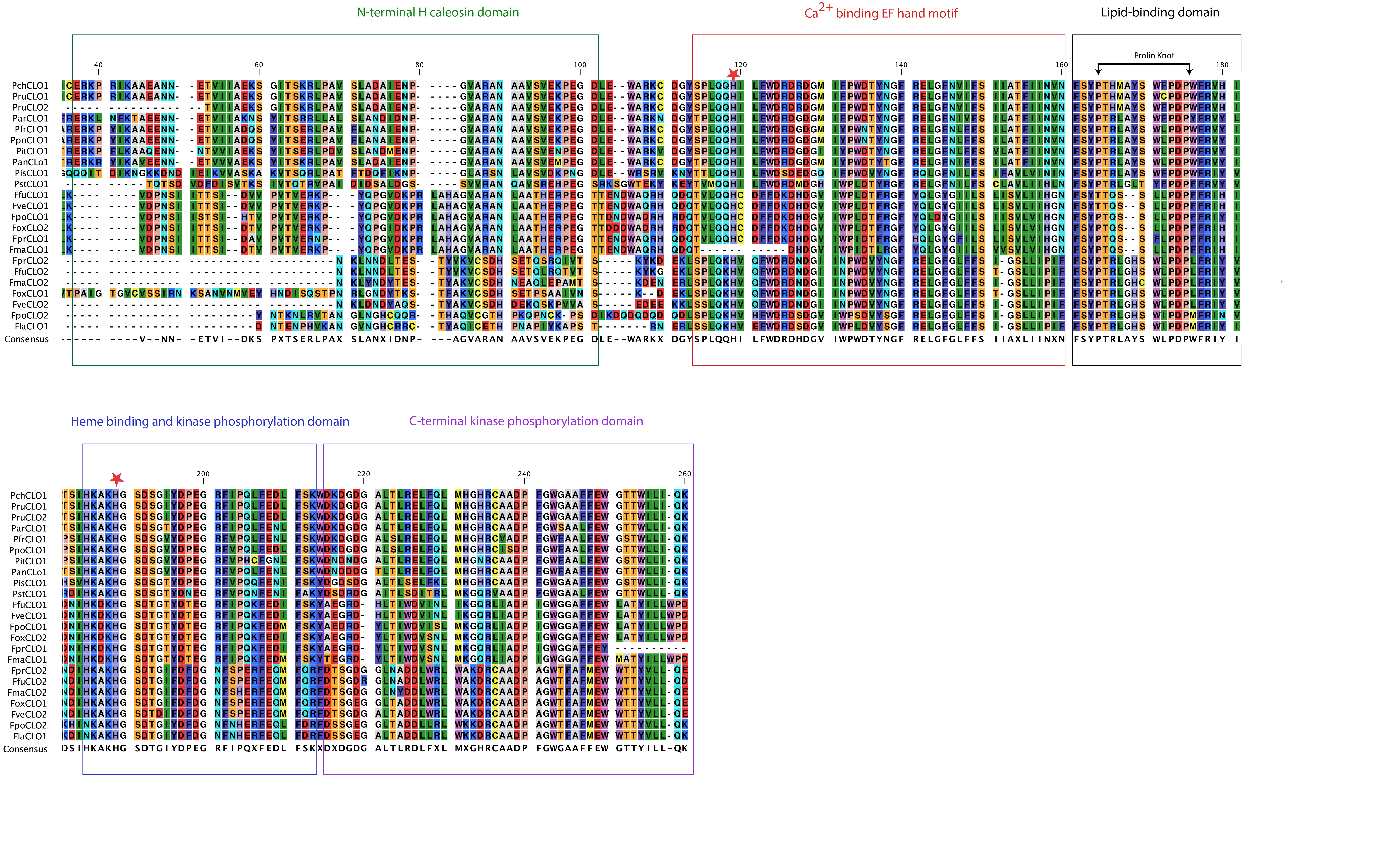

Supplement: Supplementary file 15 — Figure S2E. CLO/PXG protein sequence alignments from all sequences of Fusarium and Penicillium spp. (PNG 1349 kb) [file 12864_2018_5334_MOESM15_ESM.png]

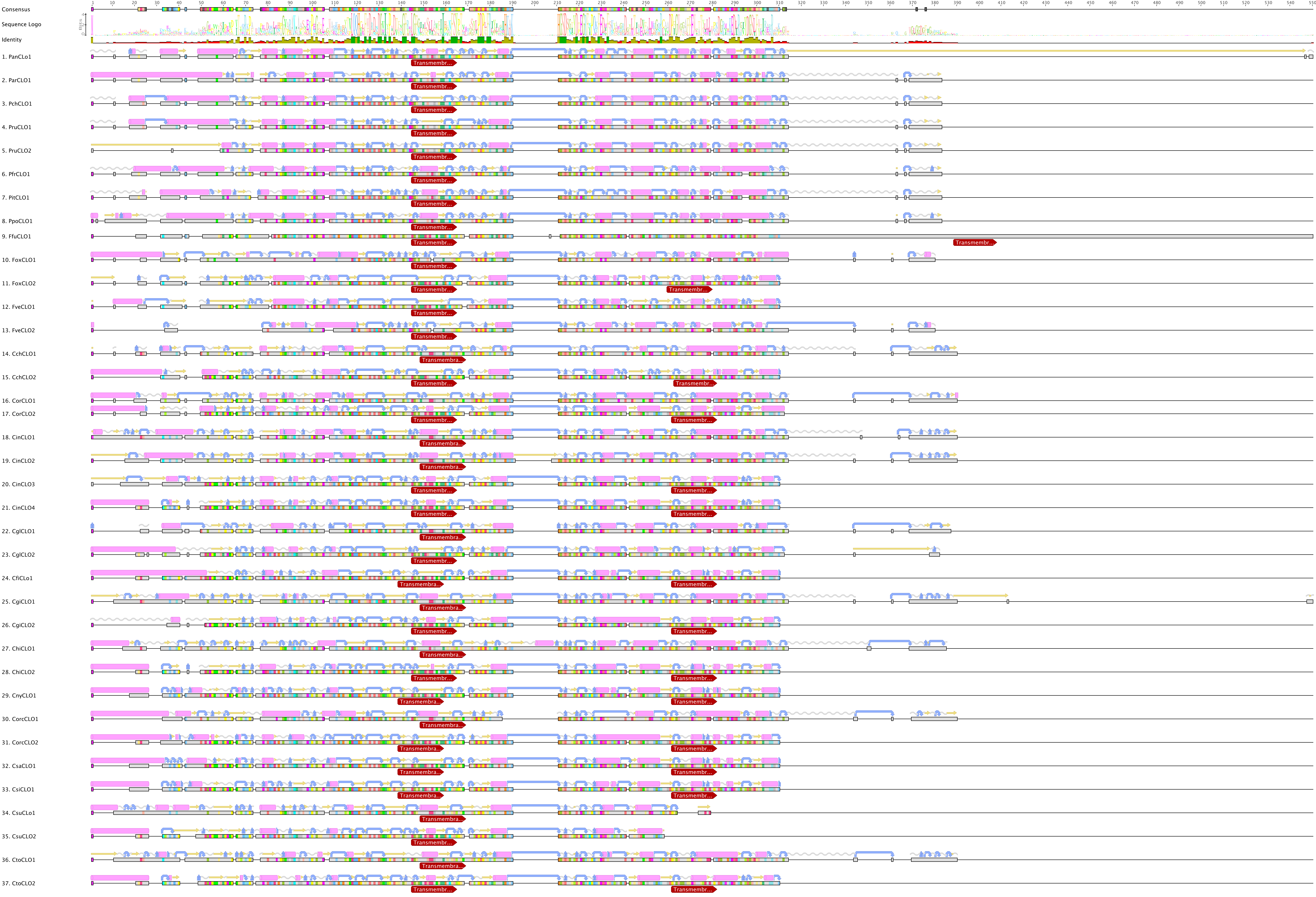

Supplement: Supplementary file 17 — Figure S3B, Predicted secondary structures of CLO/PXG proteins from Penicillium, Fusarium and Colletotrichum genera. (PDF 869 kb) [file 12864_2018_5334_MOESM17_ESM.pdf]

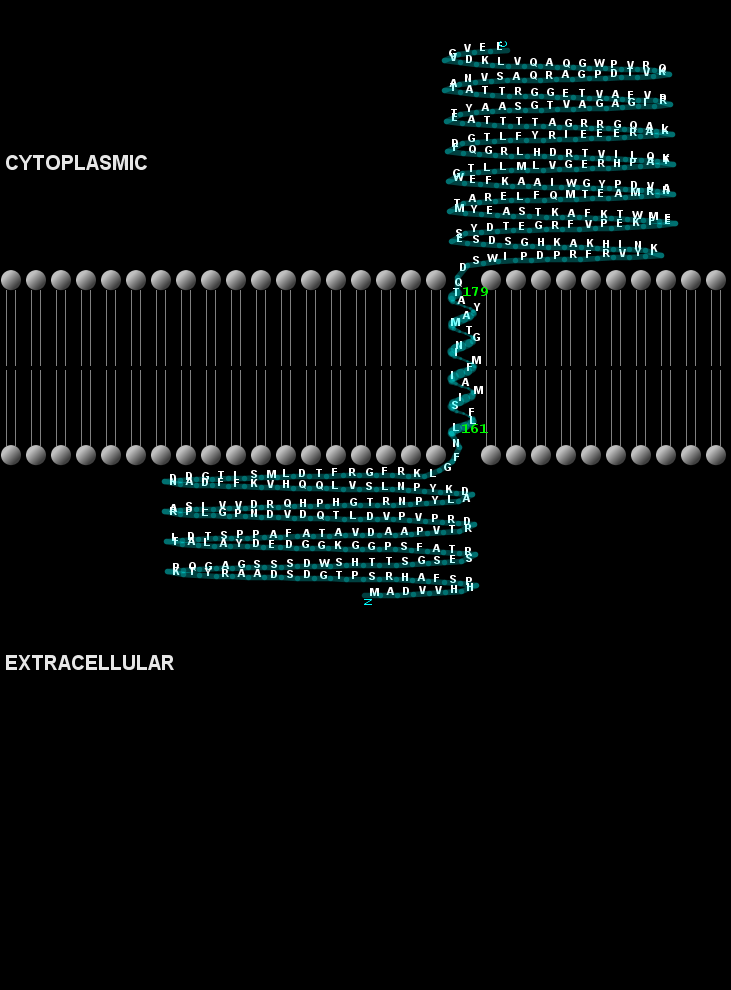

Supplement: Supplementary file 18 — Figure S3C. Predicted subcellular orientation of a fungal CLO/PXG protein with one transmembrane domain (Allomyces macrogynus). (PNG 66 kb) [file 12864_2018_5334_MOESM18_ESM.png]

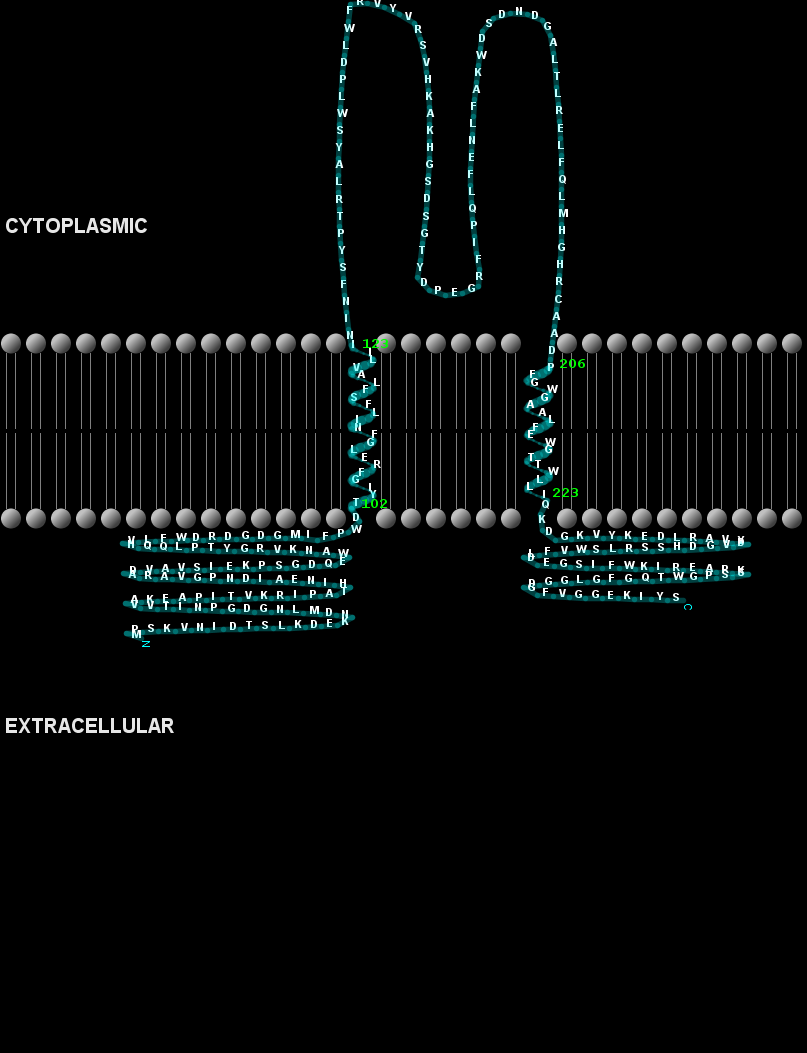

Supplement: Supplementary file 19 — Figure S3D. Predicted subcellular orientation of a fungal CLO/PXG protein with two transmembrane domains (Aspergillus flavus). (PNG 70 kb) [file 12864_2018_5334_MOESM19_ESM.png]

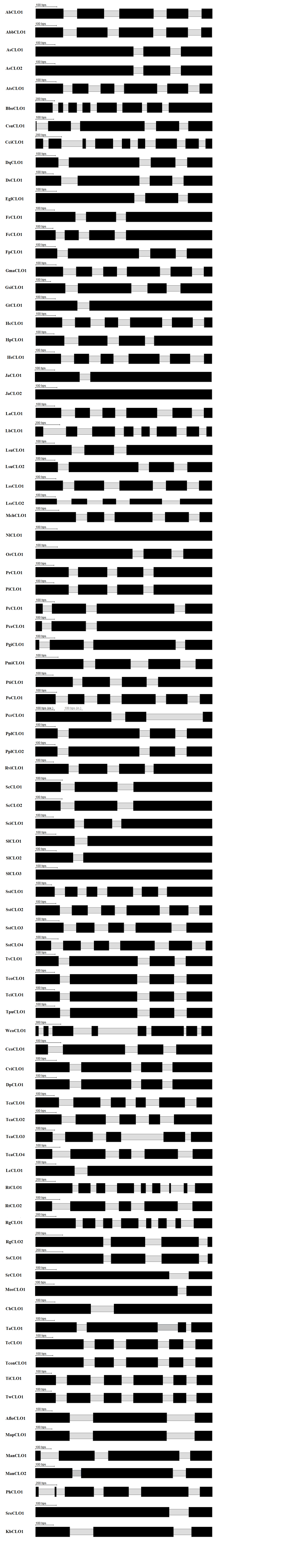

Supplement: Supplementary file 20 — Figure S4A. Predicted CLO/PXGs gene structures of first group of Basidiomycota. (PNG 268 kb) [file 12864_2018_5334_MOESM20_ESM.png]

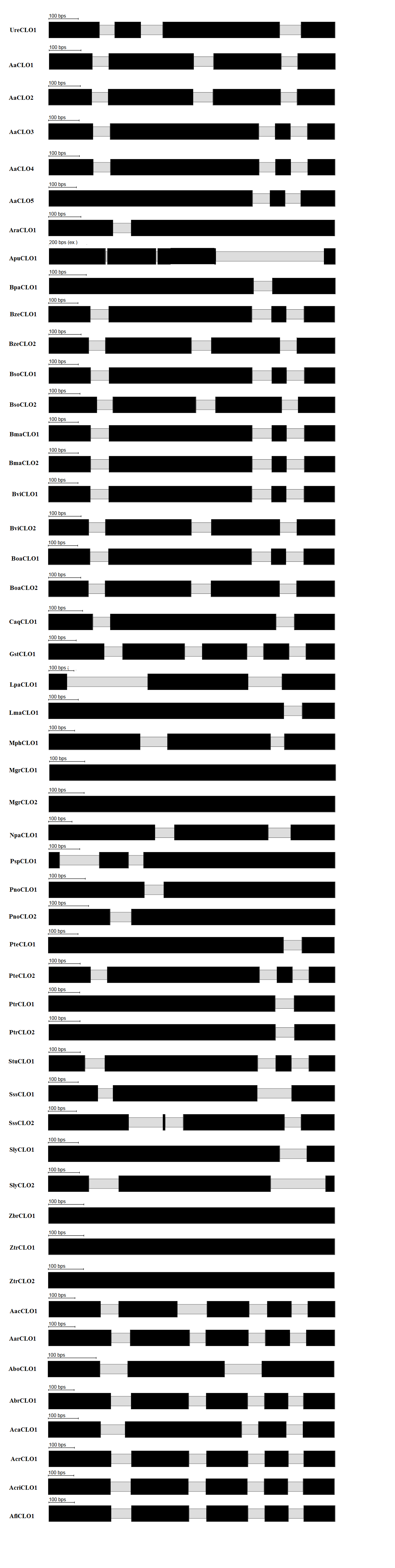

Supplement: Supplementary file 22 — Figure S4C. Predicted CLO/PXGs gene structures of first group of Ascomycota. (PNG 141 kb) [file 12864_2018_5334_MOESM22_ESM.png]

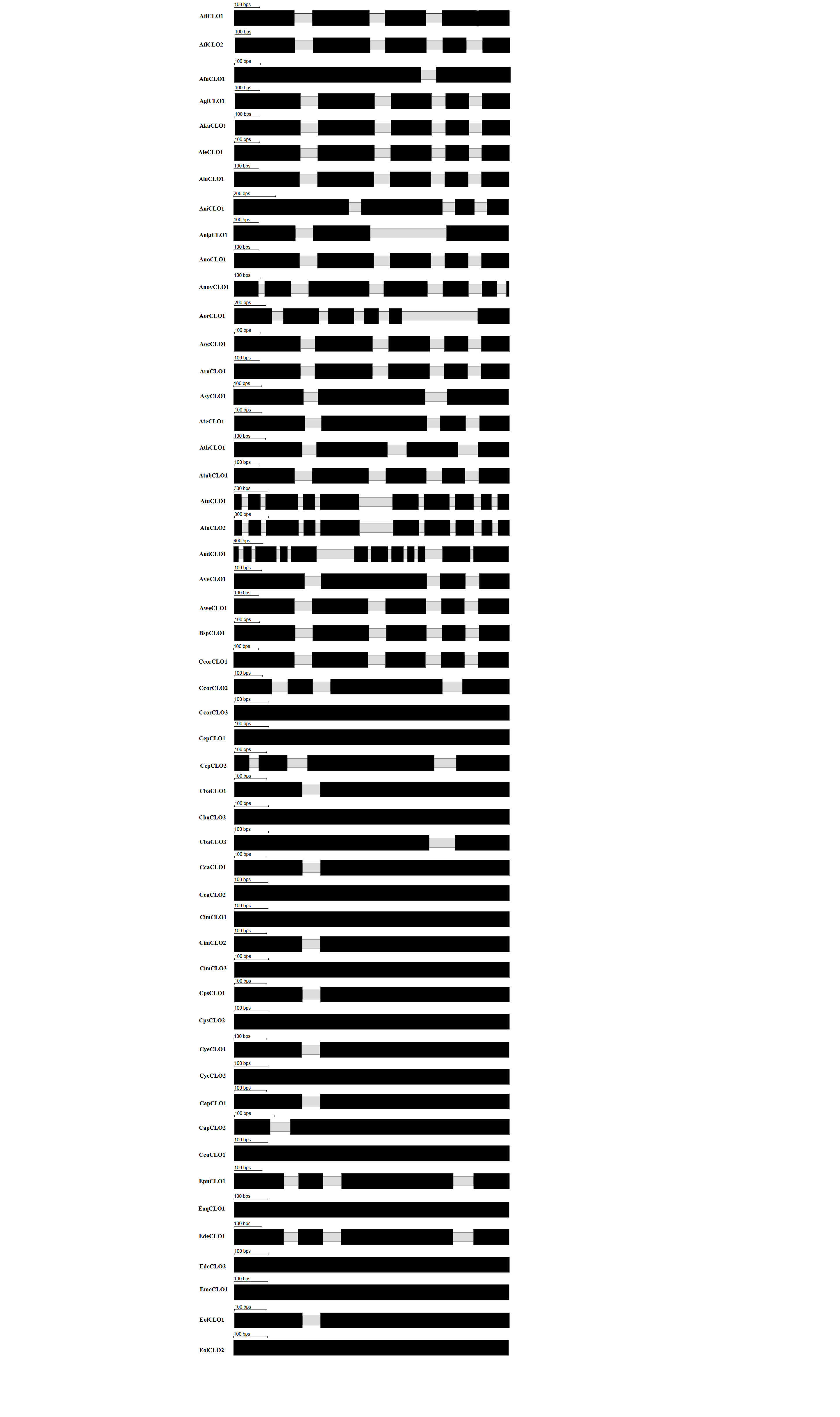

Supplement: Supplementary file 23 — Figure S4D. Predicted CLO/PXGs gene structures of second group of Ascomycota. (PNG 170 kb) [file 12864_2018_5334_MOESM23_ESM.png]

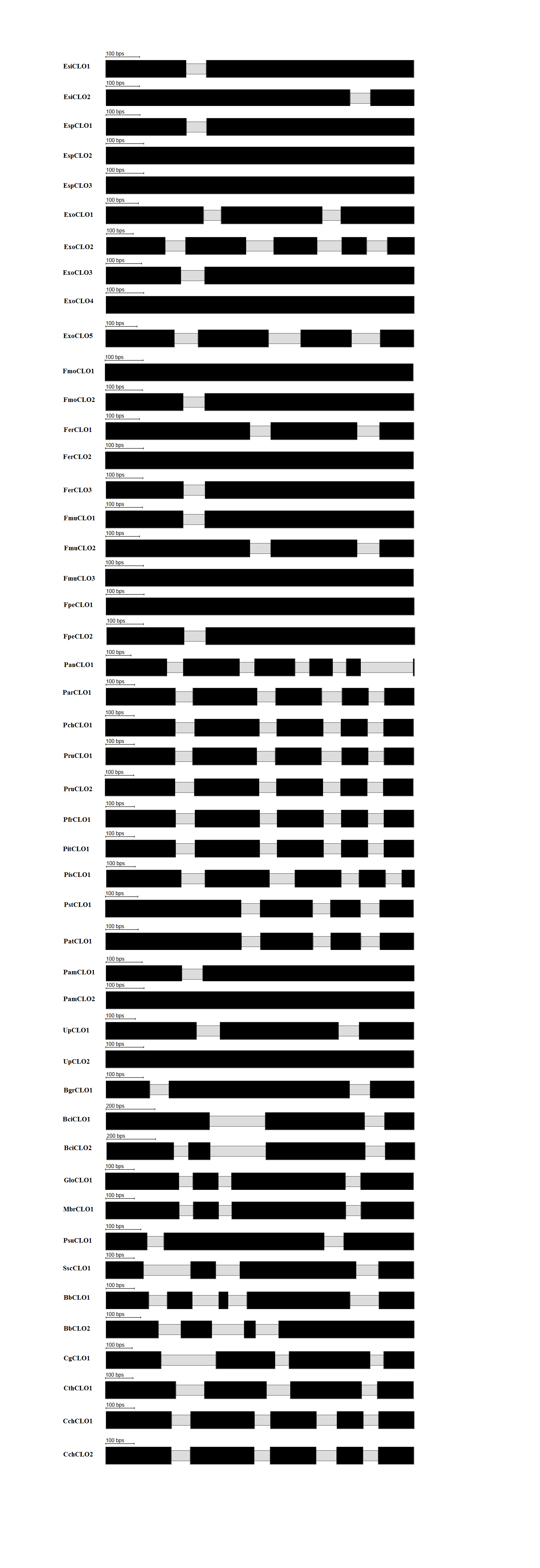

Supplement: Supplementary file 24 — Figure S4E. Predicted CLO/PXGs gene structures of third group of Ascomycota. (PNG 160 kb) [file 12864_2018_5334_MOESM24_ESM.png]

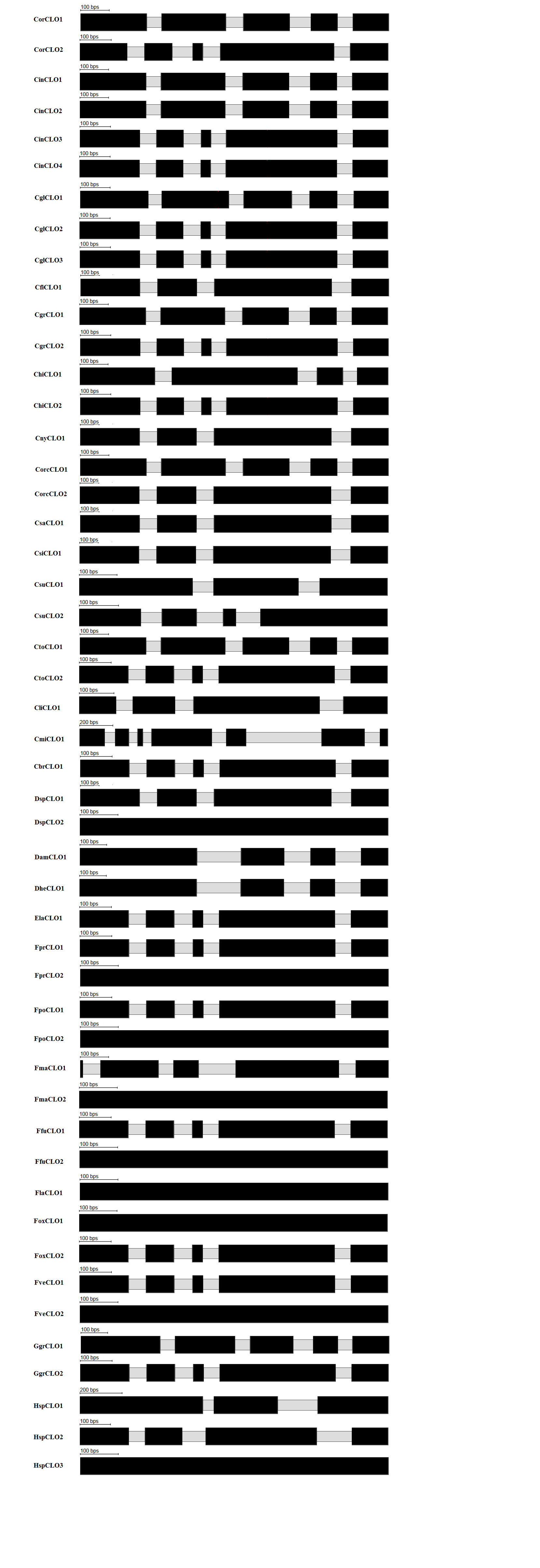

Supplement: Supplementary file 25 — Figure S4F. Predicted CLO/PXGs gene structures of fourth group of Ascomycota. (PNG 172 kb) [file 12864_2018_5334_MOESM25_ESM.png]

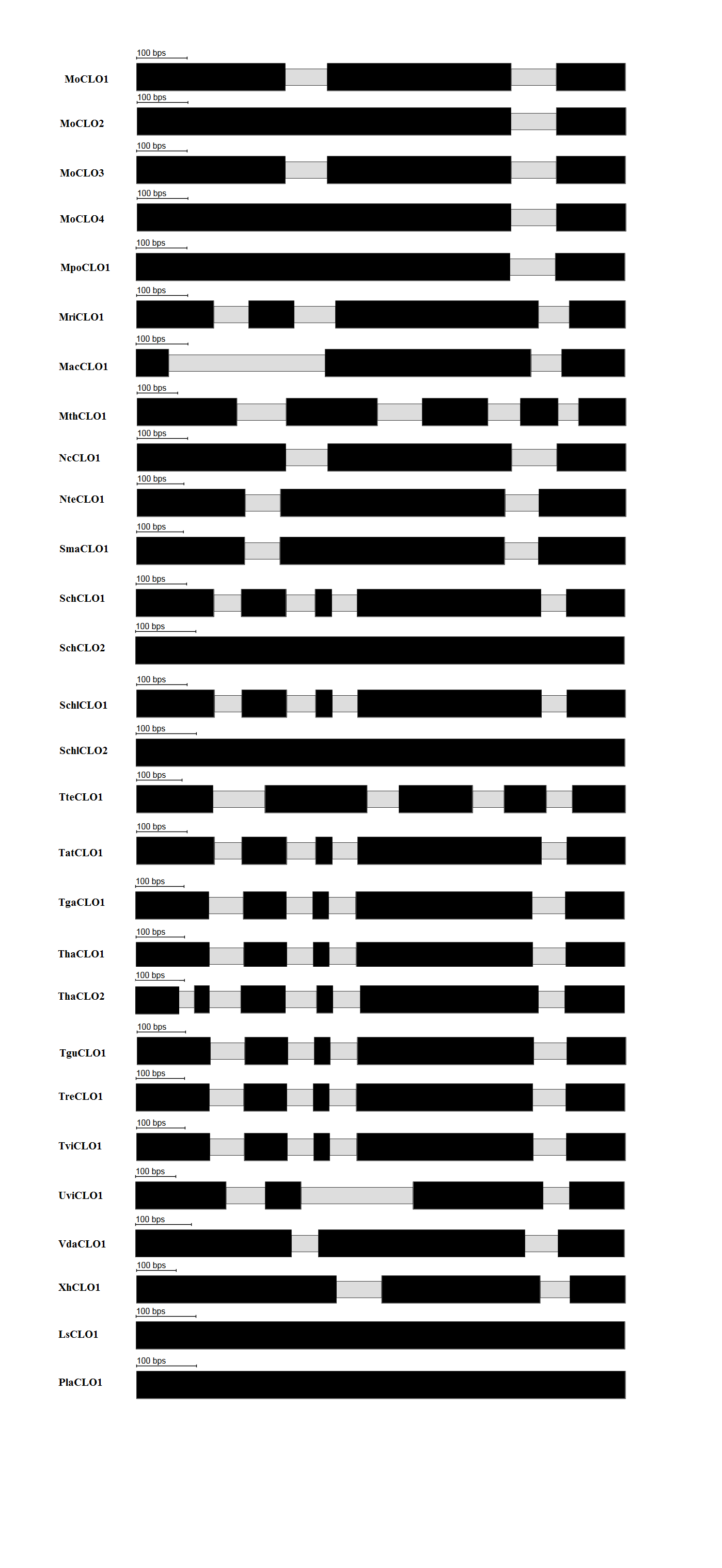

Supplement: Supplementary file 26 — Figure S4G. Predicted CLO/PXGs gene structures of fifth group of Ascomycota. (PNG 95 kb) [file 12864_2018_5334_MOESM26_ESM.png]

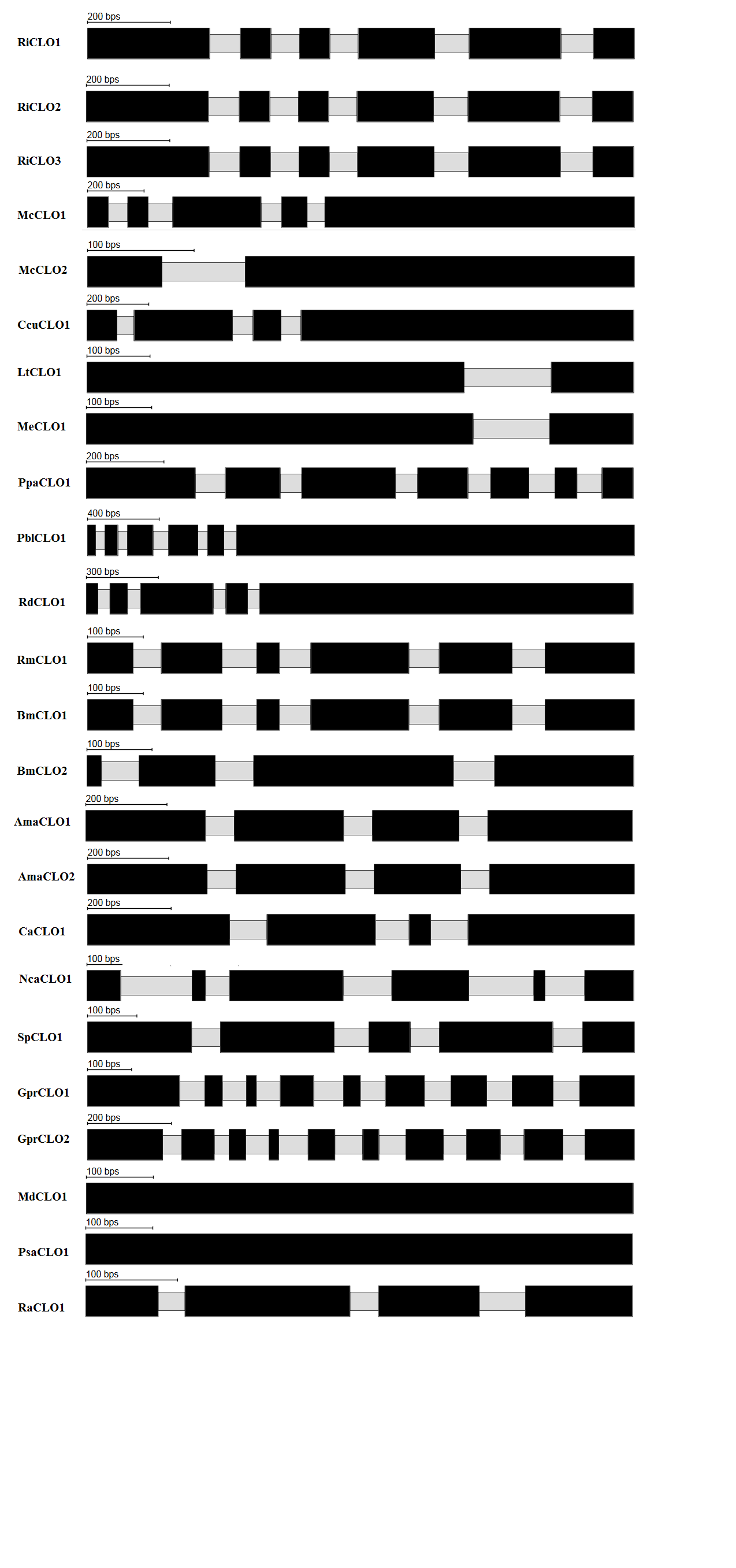

Supplement: Supplementary file 27 — Figure S4H. Predicted CLO/PXGs gene structures of non-Dikarya species. (PNG 86 kb) [file 12864_2018_5334_MOESM27_ESM.png]
