# Supplementary material for: Evolutionary, structural and functional analysis of the caleosin/peroxygenase gene family in the Fungi
Source: BMC Genomics. 2018 Dec 28;19:976. doi: 10.1186/s12864-018-5334-1 (PMC6309107; doi:10.1186/s12864-018-5334-1)
Supplement: Supplementary file 28 — Figure S5. Maximum Likelihood phylogeny for all 462 analysed plant and fungal CLO/PXG proteins. The optimum model of protein substitution was found to be LG + G. Bootstrap resampling (100 iterations) was undertaken and are shown on internal nodes. There are several strongly supported clades but support values inferring sister group relationships between these are extremely low. Species names are coloured relative to their taxonomy. The presence of MEME predicted motifs are shown for individual proteins. (PDF 5353 kb) [file 12864_2018_5334_MOESM28_ESM.pdf]

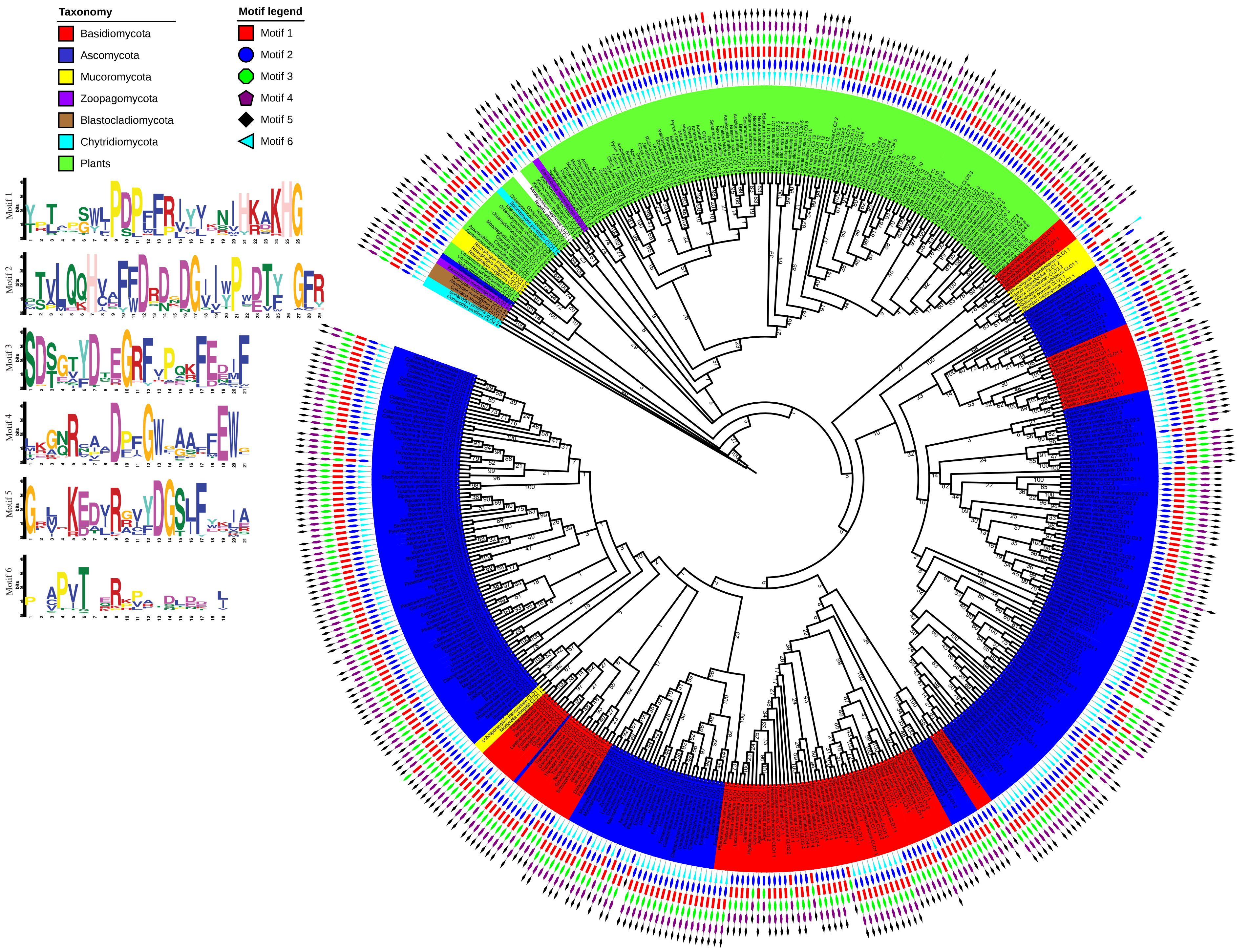

Taxonomy
Basidiomycota
Ascomycota
Mucoromycota
Zoopagomycota
Blastocladiomycota
Chytridiomycota
Plants
Motif legend
Motif 1
Motif 2
Motif 3
Motif 4
Motif 5
Motif 6
