# Supplementary material for: Evolutionary, structural and functional analysis of the caleosin/peroxygenase gene family in the Fungi
Source: BMC Genomics. 2018 Dec 28;19:976. doi: 10.1186/s12864-018-5334-1 (PMC6309107; doi:10.1186/s12864-018-5334-1)
Supplement: Supplementary file 29 — Figure S6. Phylogenetic network reconstructed using the representative 199 plant and fungal CLO/PXG proteins. The neighbour-joining network method was used to infer splits within the alignment. Species names are coloured relative to their taxonomy. Strongly supported monophyletic clades are evident within the network. However relationships between these clades is conflicting as illustrated by many alternative splits at the base of the network. (PDF 556 kb) [file 12864_2018_5334_MOESM29_ESM.pdf]

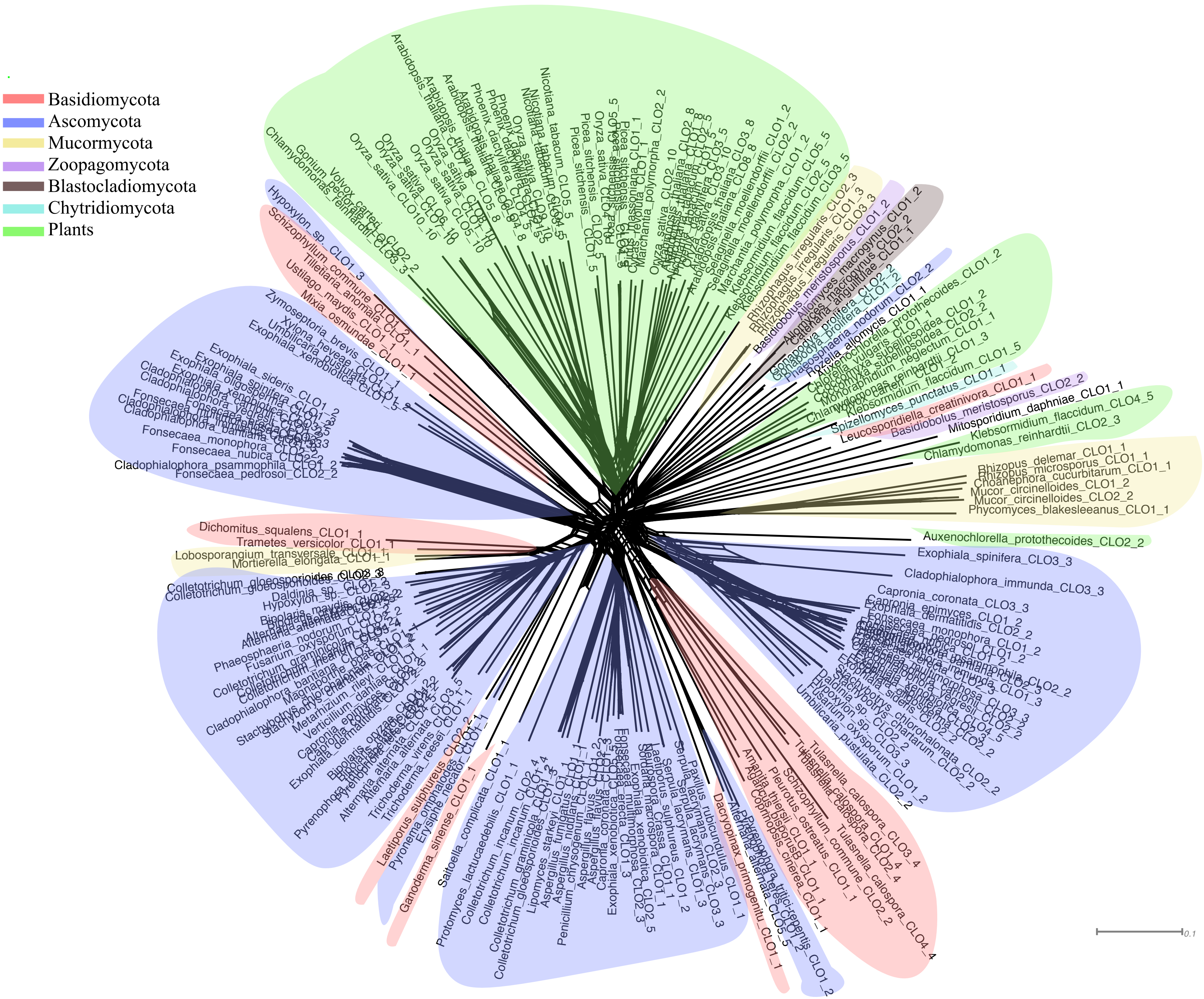
Basidiomycota
Ascomycota
Mucormycota
Zoopagomycota
Blastocladiomycota
Chytridiomycota
Plants
0.1
