# Supplementary material for: Evolutionary, structural and functional analysis of the caleosin/peroxygenase gene family in the Fungi
Source: BMC Genomics. 2018 Dec 28;19:976. doi: 10.1186/s12864-018-5334-1 (PMC6309107; doi:10.1186/s12864-018-5334-1)
Supplement: Supplementary file 12 — Figure S2B. CLO/PXG protein sequence alignments from all sequences of Ascomycota. (PDF 7080 kb) [file 12864_2018_5334_MOESM12_ESM.pdf]

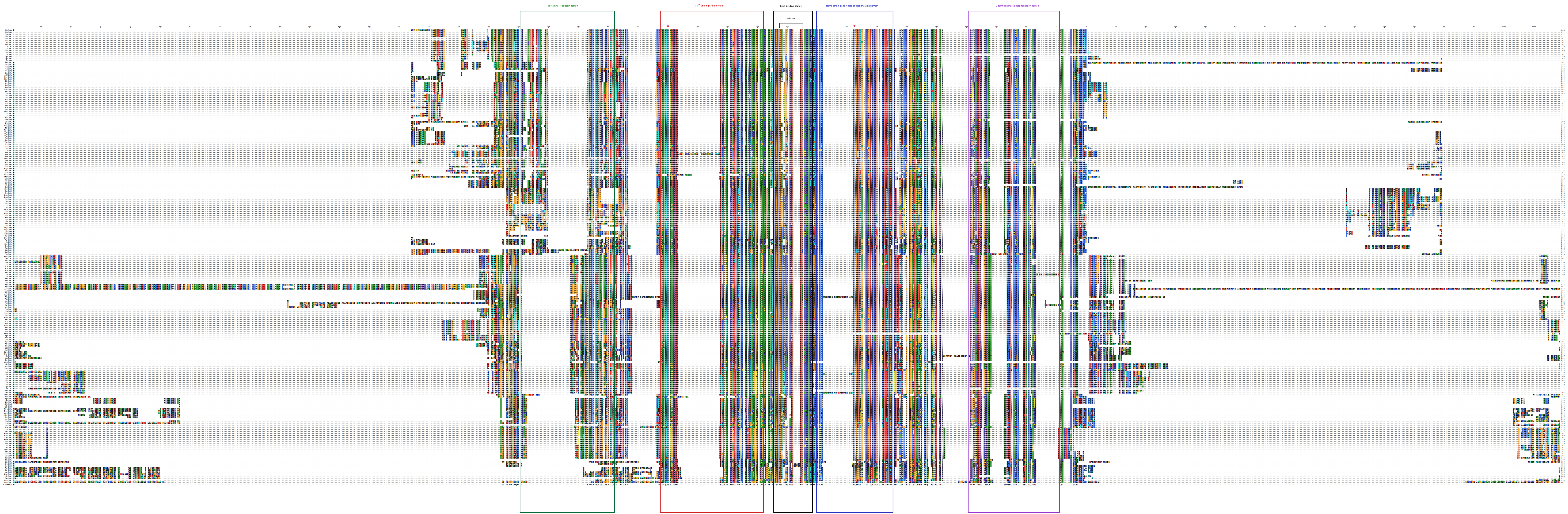

N-terminal H caleosin domain
Ca2+ binding EF hand motif
Lipid-binding domain
Proline knot
Heme binding and kinase phosphorylation domain
C-terminal kinase phosphorylation domain
